# Directed evolution of *Escherichia coli* surface-displayed *Vitreoscilla* hemoglobin as an artificial metalloenzyme for the synthesis of 5-imino-1,2,4-thiadiazoles†

**DOI:** 10.1039/d4sc00005f

**Published:** 2024-04-25

**Authors:** Yaning Xu, Fengxi Li, Hanqing Xie, Yuyang Liu, Weiwei Han, Junhao Wu, Lei Cheng, Chunyu Wang, Zhengqiang Li, Lei Wang

**Affiliations:** a Key Laboratory of Molecular Enzymology and Engineering of Ministry of Education, School of Life Sciences, Jilin University Changchun 130023 P. R. China lzq@jlu.edu.cn w_lei@jlu.edu.cn; b State Key Laboratory of Supramolecular Structure and Materials, Jilin University Changchun 130023 P. R. China

## Abstract

Artificial metalloenzymes (ArMs) are constructed by anchoring organometallic catalysts to an evolvable protein scaffold. They present the advantages of both components and exhibit considerable potential for the *in vivo* catalysis of new-to-nature reactions. Herein, *Escherichia coli* surface-displayed *Vitreoscilla* hemoglobin (VHb^SD-Co^) that anchored the cobalt porphyrin cofactor instead of the original heme cofactor was used as an artificial thiourea oxidase (ATOase) to synthesize 5-imino-1,2,4-thiadiazoles. After two rounds of directed evolution using combinatorial active-site saturation test/iterative saturation mutagenesis (CAST/ISM) strategy, the evolved six-site mutation VHb^SD-Co^ (6SM-VHb^SD-Co^) exhibited significant improvement in catalytic activity, with a broad substrate scope (31 examples) and high yields with whole cells. This study shows the potential of using VHb ArMs in new-to-nature reactions and demonstrates the applicability of *E. coli* surface-displayed methods to enhance catalytic properties through the substitution of porphyrin cofactors in hemoproteins *in vivo*.

## Abstract 2

*Escherichia coli* surface-displayed *Vitreoscilla* hemoglobin as an artificial metalloenzyme for the synthesis of 5-imino-1,2,4-thiadiazoles.

## Graphical abstract

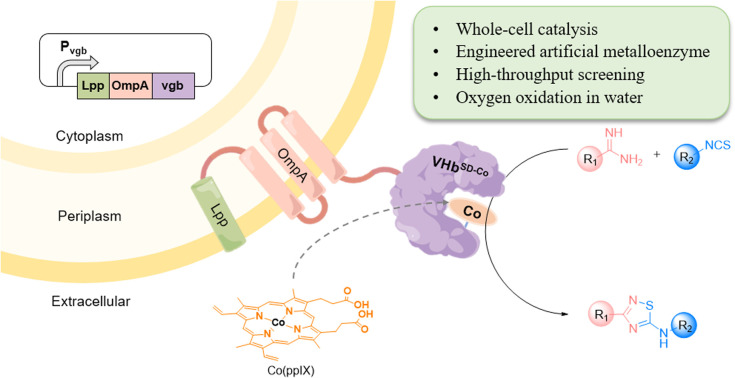

## Introduction

**Scheme 1:**
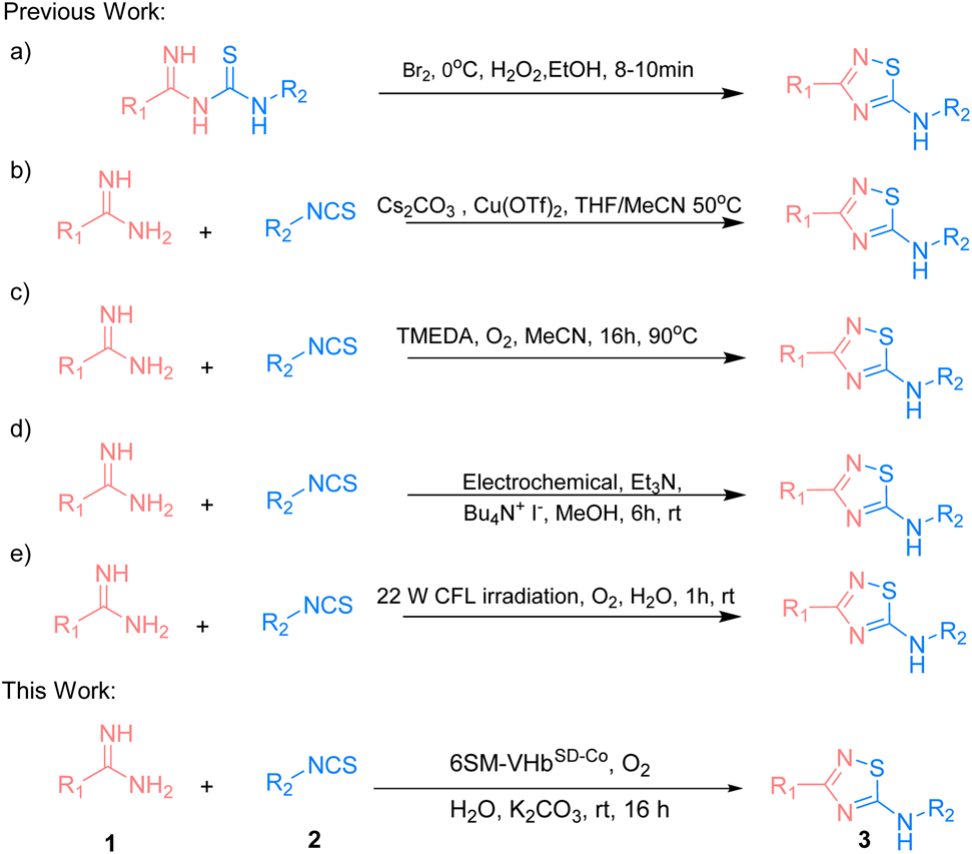
Strategies for synthesis of 5-imino-1,2,4-thiadiazole derivatives.

The 5-imino-1,2,4-thiadiazole motif is a unique unit in organic synthesis that can be easily transformed into a large number of functional groups.^1,2^ It also plays an important role in bioactive molecules.^3–5^ Existing methodologies focus on the intramolecular oxidative cyclization of imidoyl thiourea to form the corresponding 5-imino-1,2,4-thiadiazoles (Scheme 1a).^6^ In 1959, Tertiuk *et al.* proposed an intramolecular oxidative reaction from imidoyl thiourea by using Br_2_ as an oxidant at 0 °C for the synthesis of 5-imino-1,2,4-thiadiazoles.^7^ To date, several reports have been made on using stoichiometric oxidants (*e.g.*, H_2_O_2_, I_2_ or hypervalent iodine) or transition metals for the synthesis of 5-imino-1,2,4-thiadiazoles *via* the oxidative cyclization of imidoyl thiourea. In 2014, Gong *et al.* presented Cu(OTf)_2_-catalyzed imidoyl thioureas *in situ* by amidine hydrochlorides with isothiocyanates for the synthesis of 5-imino-1,2,4-thiadiazoles *via* intramolecular N–S bond formation at 50 °C (Scheme 1b).^8^ In 2019, a strategy that used the tetramethylethylenediamine-mediated intramolecular oxidative synthesis of 5-imino-1,2,4-thiadiazoles in MeCN at 90 °C was proposed (Scheme 1c).^9^ In the succeeding years, Liu *et al.* explored an electrochemical method for the synthesis of 5-imino-1,2,4-thiadiazoles through intermolecular S–N coupling (Scheme 1d).^10^ Siddiqui *et al.* employed a visible light-promoted formation of the S–N bond for the synthesis of the 5-imino-1,2,4-thiadiazole motif in EtOH : H_2_O (4 : 1) at room temperature (Scheme 1e).^11^ However, some drawbacks exist, such as the use of high temperature, harmful organic solvents, or the limitation of the substrate scope. Therefore, exploring eco-friendly, green, and efficient strategies that have not yet been realized should be given importance when constructing these valuable and highly attractive 5-imino-1,2,4-thiadiazole structures.^12–20^

Artificial metalloenzymes (ArMs) are an innovative type of biocatalysts that possess abiotic metal cofactors.^21–26^ They are promising tools for engineering new non-natural chemical transformations.^27–33^ Our group previously reported a Co(ppIX)-linked ArM that was designed based on *Vitreoscilla* hemoglobin (VHb).^34,35^ The cobalt porphyrin cofactor was incorporated into the cavity of VHb (VHb^Co^) *in vivo* by using a porphyrin synthesis-deficient *Escherichia coli* strain RP523,^36^ and a range of engineered VHb^Co^ variants were found to catalyze intramolecular oxidative cyclization for the synthesis of 2-substituted benzoxazoles/benzothiazoles.

However, many metal cofactors are inhibited by cellular components and have limited access to the cytoplasm, indicating that the scaffold protein must be purified.^37^ These factors limit the throughput of genetic optimization schemes applied to ArMs and the applicability *in vivo* to expand natural metabolism. In recent decades, numerous studies have focused on making the assembly and screening of these biohybrid catalysts more convenient: (i) *in vivo* assembly of ArM with biosynthesized cobalt protoporphyrin IX (Co(ppIX)) under iron-limited, cobalt-rich growth conditions,^38,39^ (ii) *in vivo* assembly of ArM and cofactor with a system to transport the cofactor into the cytoplasm (*e.g.*, recombinant production of heme proteins in *E. coli* strain Nissle 1917,^40,41^ ChuA^42^ and the Hug^43^ system for porphyrin transportation), and (iii) *in vivo* screening *via* cell surface display.^37,44,45^

To circumvent the limitation of the directed evolution of VHb^Co^ based on our previous studies,^34,35,46,47^ we developed a new approach that uses *E. coli* surface-displayed method. We created a platform to display VHb^Co^ on *E. coli*'s outer membrane (VHb^SD-Co^) and established a whole-cell high-throughput screening strategy based on ultraviolet (UV) absorption for the *in vivo* directed evolution of VHb^SD-Co^ with high catalytic reactivity for the synthesis of 5-imino-1,2,4-thiadiazoles. After two rounds of evolution, the best VHb^SD-Co^ mutant exhibited excellent activity, producing thiadiazole products with broad substrate scopes, and it can be further evolved in different directions by adjusting the workflow. Our study provides a case for the systematic implementation and directed evolution of an ArM that can be applied to an *in vivo* non-natural reaction by using O_2_ as the oxidant and H_2_O as the solvent. Notably, this study is the first successful attempt to catalyze the synthesis of 1,2,4-thiadiazoles by using a biocatalyst, and it exhibits considerable potential for the further exploration of other non-natural reactions *in vivo*.

## Result and discussion

Our study started with the reaction of benzamidine (1a) and phenyl isothiocyanate (2a) under aerobic conditions at room temperature to form the 5-imino-1,2,4-thiadiazole compound 3aa catalyzed by wild-type VHb. Apart from VHb, several hemeproteins and ArMs that were reported in our previous works were also evaluated (Table 1).^34,35^ The synthesis of 3aa could not proceed without a catalyst, as shown in entry 1. Cytochrome C, myoglobin, and horseradish peroxidase exhibited low reactivity, while hemoglobins catalyzed the formation of 3aa with better yields (entries 2–10). Among all of these hemeproteins, VHb presented the highest reactivity. Similar to the oxidation reactions reported in previous works, VHb^Co^ demonstrated promising activity compared with wild-type VHb and VHbArM with Mn(ppIX) (VHb^Mn^) and Zn(ppIX) (VHb^Zn^) (entries 11–13). Therefore, VHb^Co^ was selected to be the best biocatalyst for further study.

**Table 1:** Biocatalysts screening for the synthesis of 3aa^a^

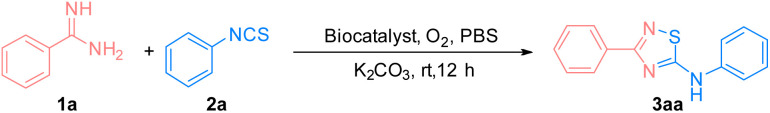			
Entry	Biocatalysts	Yield^b^ (%)	TON
1	—	0	0
2	VHb	38	760
3	Myoglobin (equine skeletal muscle)	20	400
4	Hemoglobin (human)	22	440
5	Hemoglobin (rabbit)	25	500
6	Hemoglobin (bovine)	26	520
7	Cytochrome C (horse heart)	15	300
8	Cytochrome C (porcine heart)	14	280
9	Cytochrome C (bovine heart)	20	400
10	Horseradish peroxidase	19	380
11	VHb^Co^	49	980
12	VHb^Mn^	17	340
13	VHb^Zn^	Trace	Trace

a Reaction condition: benzamidines (1a, 50 mM), phenyl isothiocyanate (2a, 50 mM), catalyst (metalloporphyrin containing 0.05 mol%), K_2_CO_3_ 30 mM, O_2_, PBS buffer (10 mM), stirred at rt for 12 h.

b Determined by high-performance liquid chromatography (HPLC).

**Fig. 1:**
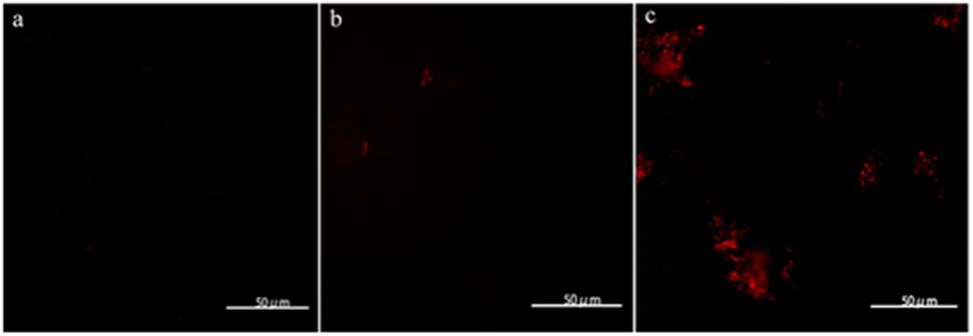
Fluorescence microscopy of immuno-stained *E. coli* cells. (a) *E. coli* cells without VHb, (b) *E. coli* cells expressing VHb^Co^ in the cytoplasm, (c) *E. coli* cells expressing VHb^SD-Co^ on the surface. Cells were labeled with a primary mouse anti-6xhistag-antibody followed by a fluorescently-labeled secondary goat-anti-mouse antibody.

Considering that the intracellular assembly of VHb^Co^ is inefficient for mutant screening and larger substrates experience difficulty in entering *E. coli* cells, we developed a VHb^Co^ surface display platform on the outer membrane of *E. coli* by using Lpp–OmpA anchor. By fusing truncated *E. coli* lipoprotein Lpp, the first five β-strands of outer membrane protein OmpA, a Gly×5 linker, and VHb protein (with His×6 tag on the C-terminus), VHb was anchored onto the outer membrane. To demonstrate the successful construction of Lpp–OmpA–VHb fusion ArM (VHb^SD-Co^), *E. coli* cells were stained with a primary mouse-anti his-tag antibody after expression and labeled with a secondary fluorescent antibody, followed by fluorescence microscopy analysis (Fig. 1). Antibodies could not easily cross the membrane and stain the VHb^Co^ protein expressed in the cytoplasm as we anticipated (Fig. 1b). The control experiment of *E. coli* cells without VHb protein revealed the fluorescence labeling of *E. coli* only in the presence of His×6 tag-labeled VHb displayed on the surface of the outer membrane (Fig. 1a and c). As expected, these results were consistent with previous reports of Lpp–OmpA-labeled proteins.^37,44,45,48,49^

To test the catalytic activity of VHb^SD-Co^, the fusion protein was expressed in *E. coli* cells under anaerobic conditions. The cells were spun-down, and the pellets were resuspended in substrate buffer (1a 50 mM, 2a 50 mM, and K_2_CO_3_ 30 mM). Then, the reaction mixture was incubated at room temperature for 12 h. The yield of the reaction was determined *via* high-performance liquid chromatography. As indicated in Table 2, purified fusion protein (entry 1) and cell lysis (entry 2) exhibited similar performance to VHb^Co^. The reaction catalyzed by VHb^SD-Co^ on the whole cells demonstrated a noticeable enhancement in yield and turnover numbers (TON) (entry 3). To further demonstrate the feasibility of VHb^SD-Co^ catalyzed in whole cells, a series of optimization experiments was carried out (Tables S1† and 2), and the results indicated that VHb^SD^ scaffold, Co(ppIX) cofactor, O_2_, K_2_CO_3_ (for the neutralization of HCl in amidine salt), and aqueous solvent played essential roles in this biocatalysis method. The optimized conditions that led to a slight increase in yield and TON of 3aa (71%, 1420 TON) are as follows: when O_2_ is used as oxidant under aerobic conditions, VHb^SD-Co^ catalyzes the synthesis of 3aa from 1a (50 mM) and 2a (50 mM) with K_2_CO_3_ (30 mM) as base in phosphate-buffered solution (PBS) (5% MeCN for the solubilization of substrates) for 16 h.

**Table 2:** Optimization of reaction conditions^a^

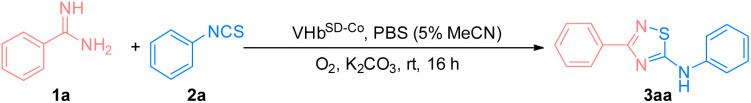				
Entry	Catalyst	Base	Yield^b^ (%)	TON
1	VHb^SD-Co^ (purified)	K_2_CO_3_	53	1060
2	VHb^SD-Co^ (cell lysis)	K_2_CO_3_	47	940
3	VHb^SD-Co^ (whole cell)	K_2_CO_3_	71	1420
4	VHb^Co^ (whole cell)	K_2_CO_3_	25	500
5	VHb^SD^ (whole cell)	K_2_CO_3_	45	900
6^c^	Co(ppIX) and *E.coli* cells	K_2_CO_3_	9	180
7^d^	VHb^SD-Co^ (whole cell)	K_2_CO_3_	27	540
8^e^	VHb^SD-Co^ (whole cell)	—	29	580
9	VHb^SD-Co^ (whole cell)	Na_2_CO_3_	43	860
10	VHb^SD-Co^ (whole cell)	NaHCO_3_	47	940

a Reaction condition: benzamidines (1a, 50 mM), phenyl isothiocyanate (2a, 50 mM), VHb^SD-Co^ (Co(ppIX) containing 0.05 mol%, details were showed in ESI), K_2_CO_3_ 30 mM, O_2_, PBS buffer (10 mM, 5% MeCN), stirred at rt for 16 h.

b Determined by HPLC.

c Without VHb^SD^.

d Air instead of O_2_.

e Without base.

**Fig. 2:**
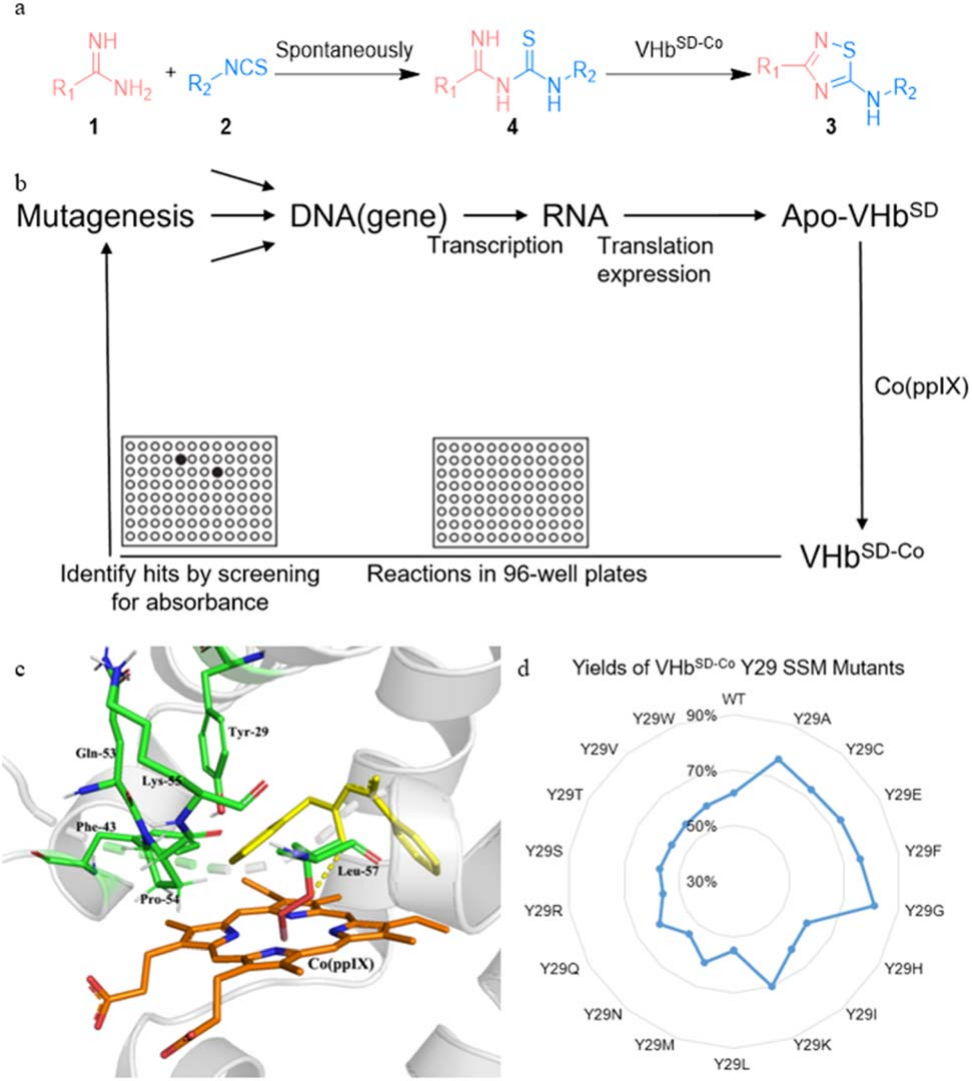
High-throughput screening of whole-cell biocatalyst VHb^SD-Co^.

We also tested several previously reported VHb^Co^ variants and mutants of axial His residue (Table S2†), but they did not perform better than the wild-type VHb^Co^ due to the different steric hindrance effects of substrates. Therefore, we developed a high-throughput screening method to select VHb^SD-Co^ variants as artificial thiourea oxidase (ATOase) with better catalytic activity. This method utilized the UV absorption discrepancy of *N*-(phenylcarbamothioyl)benzimidamide intermediate 4aa and product 3aa (Fig. S1† and 2a). Intermediate 4aa presented a characteristic absorption peak at 313 nm, while product 3aa had no UV absorption at this wavelength. In accordance with previously reported studies,^50^ substrates 1a and 2a formed intermediate 4aa spontaneously, enabling us to screen the VHb^SD-Co^ variants based on the decrease in UV absorption with reaction in 96-well plates (Fig. 2b).

To identify potential mutation sites, intermediate 4aa was docked into the structure of VHb^SD-Co^ (with Co^III^–superoxide intermediate) by using Autodock. The docking result indicated that Y29 residue on the B helix, and F43, Q53, P54, K55, and L57 residues on the loose loop region were closely related to the access of 4aa. Among them, Y29 was directly above the porphyrin plane and limited the exposure of intermediate 4aa to Co^III^–superoxide intermediate. Therefore, a combinatorial active-site saturation test/iterative saturation mutagenesis (CAST/ISM) strategy^51,52^ was applied to evolve this ATOase, the six residues were divided into two groups (Y29 and F43/Q53/P54/K55/L57) according to the region they located. At first, a site saturation mutagenesis (SSM) library at Y29 was generated, and VHb^SD-Co^ mutants performed significant changes compared with WT VHb^SD-Co^. Three better VHb^SD-Co^ single mutants were identified in the surface-displayed whole-cell screening and the yield was reevaluated separately (yield of 3aa: VHb^SD-Co^ Y29G, 81%; VHb^SD-Co^ Y29A, 77%; VHb^SD-Co^ Y29F, 76%). On the basis of the best single-site mutation VHb^SD-Co^ Y29G (defined as 1SM-VHb^SD-Co^), a CAST library of F43, Q53, P54, K55, and L57 residues was created. After this high-throughput screening, several hits were identified (Table S3†) and the best six-site mutation, VHb^SD-Co^ Y29G–F43P–Q53P–P54G–K55L–L57A (defined as 6SM-VHb^SD-Co^), was reevaluated and afforded an improved activity (yield of 3aa: 93%), and the SDS-PAGE verification was showed in Fig. S2.†

The substrate scope of amidines (1) and isothiocyanates (2) was explored using the best ATOase (6SM-VHb^SD-Co^), and the generality of this biocatalytic method for the synthesis of thiadiazoles was investigated. The results indicated that this biocatalyst was compatible with a range of amidines and isothiocyanates under standard conditions. As shown in Table 3, both electron-donating groups and electron-withdrawing groups on the phenyl groups of amidines (1) were applicable to the 6SM-VHb^SD-Co^ (3ba–3la) and afforded excellent yields (85–96%). Benzamidine with a large spatial-resistance group showed a decrease on the reactivity, it might because the biphenyl benzamidine is a big substrate which is harder to access into the cavity of VHb, and also probably dispersed into the membrane due to the less water solubility (3ha). Meanwhile, small substituents on the phenyl group of substrates 1 do not lead to an increase in reactivity. This phenomenon may be due to the imperfect matching of intermediate 4 and the active cavity providing the worse catalytic performance based on the neighborhood and orientation effects of the enzyme. The yield of product 3ma–3oa presented varying enhancement by replacing 6SM-VHb^SD-Co^ with 1SM-VHb^SD-Co^ (the mutant with smaller substrate cavity).

**Table 3:** Substrate scope of amidines (1)^a^

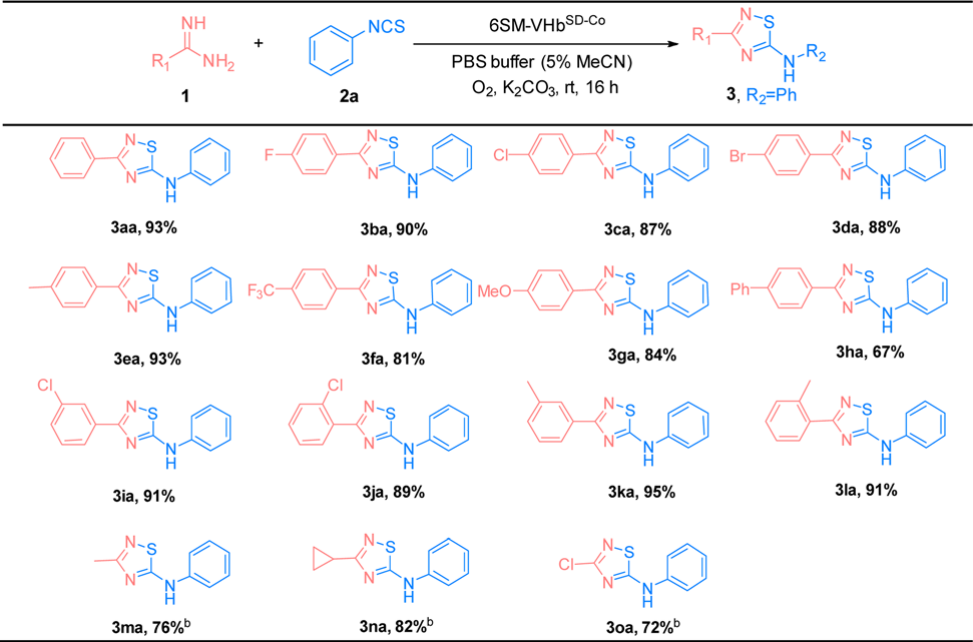

a Reaction condition: benzamidines (1a, 0.5 mmol), phenyl isothiocyanate (2a, 0.5 mmol), 6SM-VHb^SD-Co^ (Co(ppIX) containing 0.05 mol%, details were showed in ESI), K_2_CO_3_ 0.3 mmol, O_2_, PBS buffer (10 mM, 5% MeCN), stirred at rt for 16 h. Isolated yield.

b Catalyzed by 1SM-VHb^SD-Co^.

Thereafter, the scope of isothiocyanates was investigated and demonstrated a similar pattern to amidines (Table 4). The steric effects influenced the overall yield of thiadiazoles as demonstrated by the *ortho*- and *para*-substituted phenyl isothiocyanates producing decreased yields of products, compared with their *meta*-substituted counterparts (3ca, 3ia, and 3ja; 3ea, 3ka, and 3la). Encouraged by the successful results, we focused on the further application of this evolved ATOase. A preparation-scale reaction (0.5 mmol) for product 3aa was performed and afforded a satisfactory yield (95%).

**Table 4:** Substrate scope of isothiocyanates (2)^a^

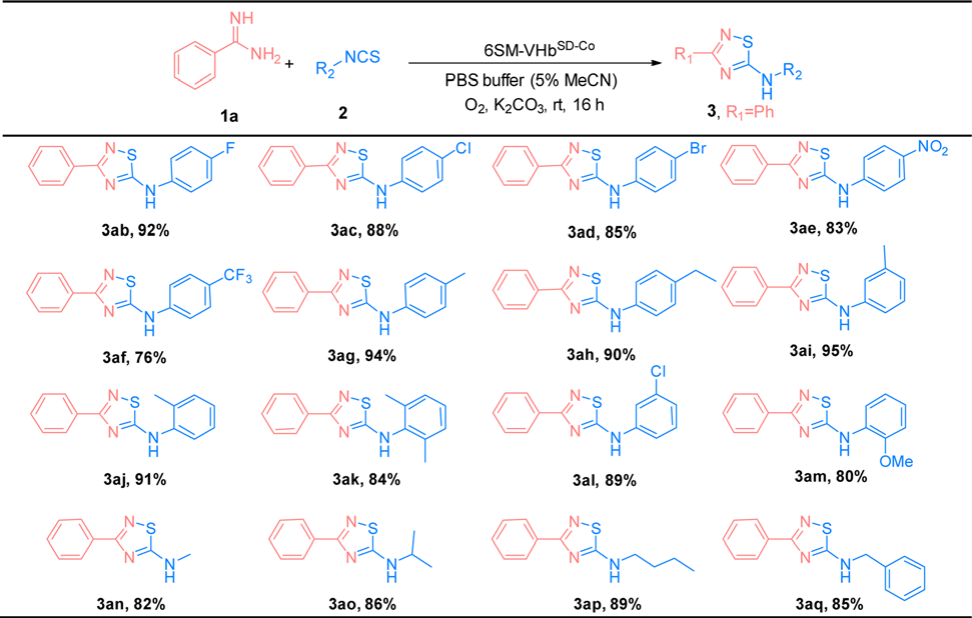

a Reaction condition: benzamidines (1a, 0.5 mmol), phenyl isothiocyanate (2a, 0.5 mmol), 6SM-VHb^SD-Co^ (Co(ppIX) containing 0.05 mol%, details were showed in ESI), K_2_CO_3_ 0.3 mmol, O_2_, PBS buffer (10 mM, 5% MeCN), stirred at rt for 16 h. Isolated yield.

To clarify the mechanism of this reaction, some control experiments were performed under specific conditions (Table 5). The results indicated that without ATOase 6SM-VHb^SD-Co^, substrates 1a and 2a coupled and converted into intermediate 4aa spontaneously (entry 1). With the addition of ATOase, intermediate 4aa afforded 3aa in 98% yield (entry 2). When the reaction was conducted in the presence of radical scavenger 2,2,6,6-tetramethylpiperdine-1-oxide (TEMPO), the yield of 3aa decreased dramatically, implying the involvement of a radical pathway in this ATOase-catalyzed reaction (entry 3). The control experiments entries 4 and 5 demonstrated the essential role of the O_2_ and Co(ppIX) cofactor of ATOase.

**Table 5:** Control experiments^a^

Entry	Conditions	Yield^b^ (%)	
1	Without VHb^SD-Co^	4aa	86
2	Using 4aa as substrate	3aa	98
3	TEMPO (3 equiv.)	3aa	Trace
4	N_2_ atmosphere	3aa	Trace
5	VHb^SD^ without Co(ppIX)	3aa	Trace

a Standard reaction condition: benzamidines (1a, 50 mM), phenyl isothiocyanate (2a, 50 mM), VHb^SD-Co^ (Co(ppIX) containing 0.05 mol%, details were showed in ESI), K_2_CO_3_ 30 mM, O_2_, PBS buffer (10 mM, 5% MeCN), stirred at rt for 16 h.

b Determined by HPLC.

**Scheme 2:**
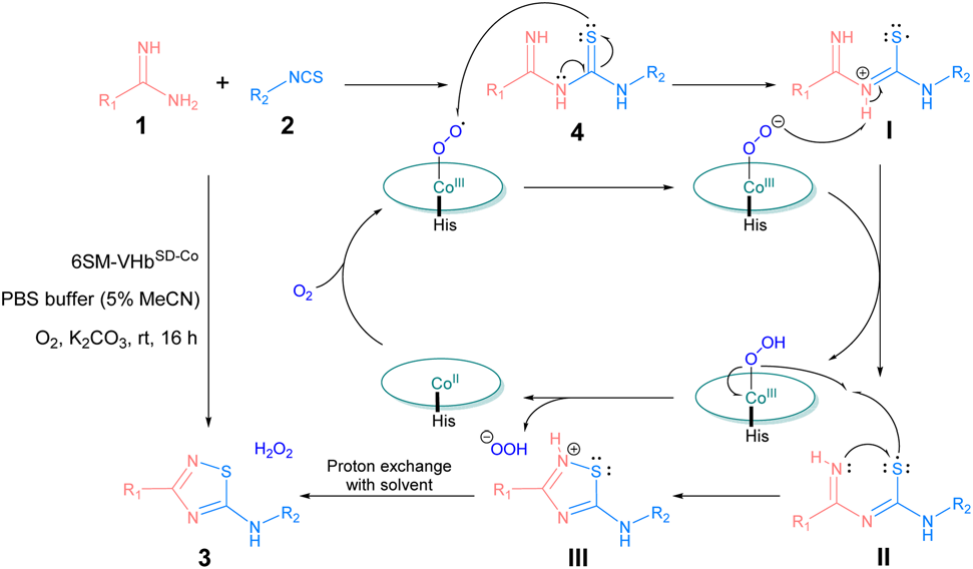
Proposed mechanism of VHb^SD-Co^-catalyzed synthesis of 1,2,4-thiadiazole.

On the basis of the previous literature^10,53,54^ and our earlier study on VHb^Co^-catalyzed aerobic oxidation,^34,35^ the mechanism of this reaction was proposed as shown in Scheme 2. First, amidines (1) and isothiocyanates (2) were coupled spontaneously to generate thiourea intermediate 4. Under aerobic conditions, the Co(ppIX) cofactor interacted with O_2_ to generate a Co^III^–superoxide intermediate, which acidified 4 to form a thiyl radical I through an electron transfer process and converted to a base itself, and then thiyl radical I transformed to intermediate II after proton transfer. Next, the nucleophilic attack on the sulfur atom by imino nitrogen led to cyclization to form intermediate III, which was accompanied by a proton transfer to Co^III^–superoxide intermediate and generated a peroxo anion. After proton exchange with solvent, III converted into 5-imino-1,2,4-thiadiazole product 3 and H_2_O_2_ was generated simultaneously which could also facilitate this reaction (the generation of H_2_O_2_ was shown in Fig. S3†).

**Fig. 3:**
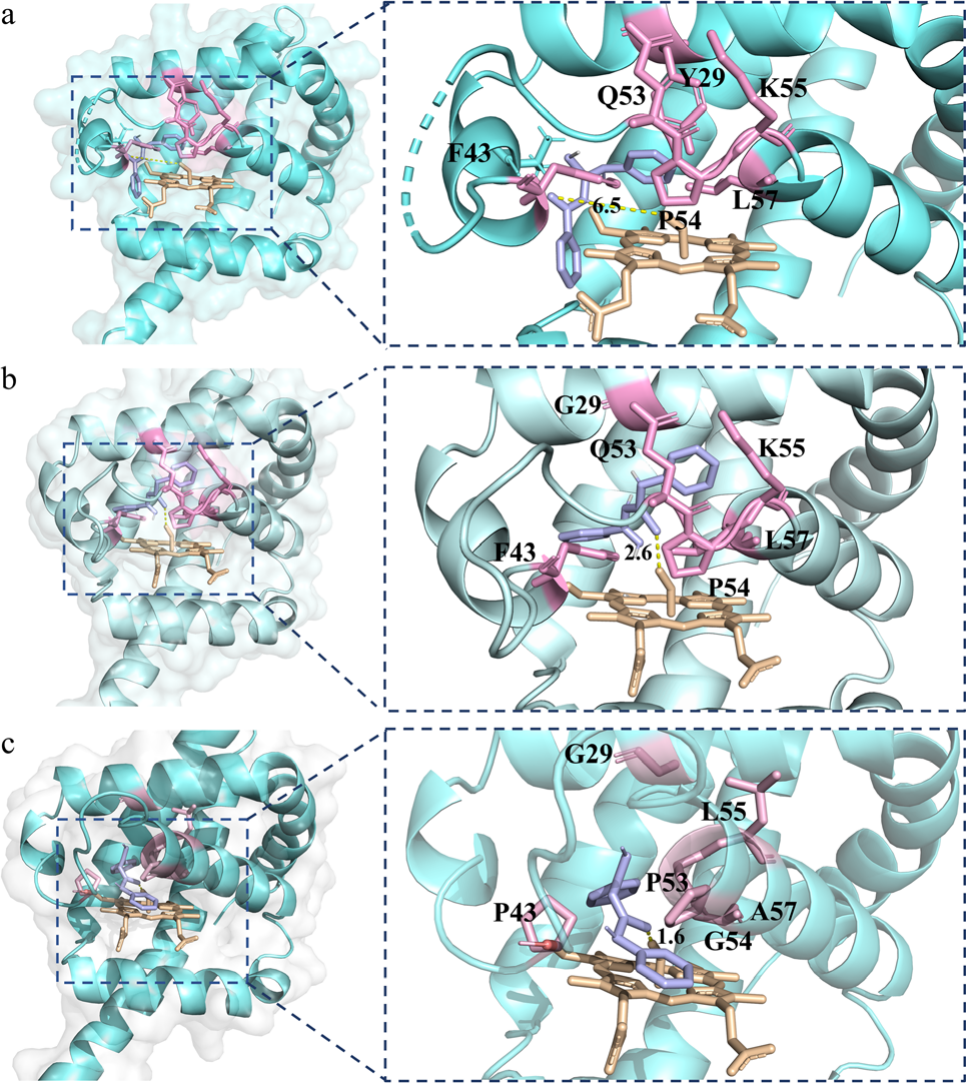
Structure model of thiourea intermediate 4aa (light purple) in the active site of VHb^SD-Co^. (a) Structure of WT-VHb^SD-Co^ in complex with 4aa. (b) Structure of 1SM-VHb^SD-Co^ in complex with 4aa. (c) Structure of 6SM-VHb^SD-Co^ in complex with 4aa.

To further explain the reactivity enhancement and catalytic mechanism of VHb^SD-Co^, thiourea intermediate (4aa) was docked into the active cavity of WT-VHb^SD-Co^, 1SM-VHb^SD-Co^ and 6SM-VHb^SD-Co^ by using the AutoDock Vina tool^55^ in Chimera.^56^ On the basis of the highest scored structures of the docking, Fig. 3 depicts the interactions of intermediate 4aa with VHb^SD-Co^, 1SM-VHb^SD-Co^ and 6SM-VHb^SD-Co^ (the structure of 1SM-VHb^SD-Co^ and 6SM-VHb^SD-Co^ were constructed by homology modeling strategy using SWISS-MODEL^57^). In particular, intermediate 4aa was accessed in a hydrophobic pocket in 6SM-VHb^SD-Co^ and well-accommodated into the active center by van der Waals contacts between proximate residues (F28, G29, F33, P43, D44, L55 and A58) and the thiourea intermediate, residue G54 stabilized intermediate by forming a hydrogen bond with the imino nitrogen on the intermediate, meanwhile, pi–alkyl interactions (L32, A57 and V98) and amide-pi stacked interactions (P53) also contributes to the approach of the intermediate (a 2D diagram showed by Discovery Studio Visualizer 4.0 (ref. 58) in Fig. S4†). The sulfur atom on intermediate 4aa maintains a slightly far distance from the oxygen atom on Co^III^–superoxide intermediate in WT-VHb^SD-Co^ (Fig. 3a), and the mutation of six amino acids with shorter side chains (Gly, Ala) and rigid side chains (Pro) adjusts the conformation of the flexible loop region (CD spectrum of WT-VHb^SD-Co^ and 6SM-VHb^SD-Co^ were showed in Fig. S5†), reduces spatial resistance to the intermediate, and brings the distance between intermediate 4aa and the oxygen atom in the Co^III^–superoxide intermediate closer in mutant 6SM-VHb^SD-Co^ (Fig. 3c).

## Conclusions

We evolved an artificial thiourea oxidase based on the porphyrin substitution of VHb. The oxidase catalyzed amidines (1) and isothiocyanates (2) to form 5-imino-1,2,4-thiadiazoles (3). To simplify the protocol of ArM construction and the screening of directed evolution, we implemented an *E. coli* surface-displayed method by using Lpp–OmpA–VHb fusion protein. Through a CAST/ISM evolution of Y29, F43, Q53, P54, K55, and L57 residues, we obtained a six-site mutation 6SM-VHb^SD-Co^ (Y29G–F43P–Q53P–P54G–K55L–L57A) with improved ATOase activity (up to 96% yield and 1920 TON, 2.5-fold increased *versus* WT VHb) and broad substrate scopes (31 examples). The molecular docking of ATOase and thiourea intermediate 4 further indicated the rationale of the reactivity enhancement. We expect that this surface-displayed method can be extended to streamline the construction and directed evolution of ArMs for new-to-nature reactions and *in vivo* synthetic biology explorations.

## Data availability

All the data supporting this article have been included in ESI.†

## Author contributions

Yaning Xu: conceptualization, investigation, writing – original draft. Fengxi Li: methodology, investigation. Hanqing Xie: investigation. Yuyang Liu & Weiwei Han: docking simulation. Junhao Wu: investigation. Lei Cheng: investigation. Chunyu Wang: NMR investigation. Zhengqiang Li: conceptualization, supervision, project administration, resources. Lei Wang: conceptualization, supervision, project administration, resources, writing – review & editing.

## Conflicts of interest

The authors declare no conflicts of interest.

## Supplementary Material

SC-015-D4SC00005F-s001
