# Supplementary material for: Directed evolution of Escherichia coli surface-displayed Vitreoscilla hemoglobin as an artificial metalloenzyme for the synthesis of 5-imino-1,2,4-thiadiazoles
Source: Chem Sci. 2024 Apr 25;15(20):7742–8. doi: 10.1039/d4sc00005f (PMC11110144; doi:10.1039/d4sc00005f)
Supplement: SC-015-D4SC00005F-s001 [file SC-015-D4SC00005F-s001.pdf]

Electronic Supplementary Material (ESI) for Chemical Science.
This journal is © The Royal Society of Chemistry 2024

Support information for

Directed Evolution of *Escherichia coli* Surface-displayed *Vitreoscilla* Hemoglobin as an Artificial Metalloenzyme for the Synthesis of 5-Imino-1,2,4-thiadiazoles

Yaning Xu [a], Fengxi Li [a], Hanqing Xie [a], Yuyang Liu [a], Weiwei Han [a], Han, Junhao Wu [a], Lei Cheng [a], Chunyu Wang [b], Zhengqiang Li [*a], Lei Wang [*a]

[a] Key Laboratory of Molecular Enzymology and Engineering of Ministry of Education, School of Life Sciences, Jilin University, Changchun 130023 (P. R. China)

[b] State Key Laboratory of Supramolecular Structure and Materials, Jilin University, Changchun 130023 (P. R. China)

E-mail: lzq@jlu.edu.cn; w_lei@jlu.edu.cn

Table of Contents

Materials and methods 2

Supporting Experimental Tables and Figures 5

Sequence of primers and variants 10

NMR Data of Products 11

^{1}H NMR Spectra 18

^{13}C NMR Spectra of New Products 34

Reference 37

Materials and methods

Materials.

All the chemicals and reagents were purchased from commercial suppliers (Sigma- Aldrich, Bide Pharmatech Ltd., Aladdin, Energy Chemical, TCI, Shanghai Chemical Reagent Company) and used without any further purification, unless otherwise stated. Horseradish peroxidase, Myoglobin from Equine skeletal muscle, Hemoglobin from bovine, rabbit and human blood, Cytochrome C from horse, porcine and bovine heart was purchased from Shanghai Yuan Ye Biological Technology Company. Ni-NTA Superflow resin obtained from Beijing Solarbio Science & Technology Co., Ltd. *E. coli* BL21(DE3) Competent Cell, Spin Miniprep, and Gel Extraction Kits were all obtained from Tiangen. *E. coli* strain RP523 was obtained from the *E. coli* Genetic Stock Center (CGSC) at Yale University. Primers and genes are obtained from Sangon Biotech. All reactions were carried out in oven-dried glassware with magnetic stirring. Silica gel chromatography purifications were carried out using AMD Silica Gel 60 230- 400 mesh. Thin Layer Chromatography (TLC) and preparative TLC were carried out using Merck Millipore TLC silica gel 60 F254 glass plates. Proton nuclear magnetic resonance (^{1}H NMR) spectra were recorded on a 400 MHz spectrometer in $CDCl_3$ or DMSO-d_6, carbon nuclear magnetic resonance (^{13}C NMR) spectra were recorded on 101 MHz spectrometer in DMSO-d_6. Chemical shifts for protons are reported in parts per million downfield from tetramethylsilane (TMS) and are referenced to residual protium in the NMR solvent ($CHCl_3$ = δ 7.26 ppm, DMSO-d_6 = δ 2.50 ppm). Chemical shifts for carbon are reported in parts per million downfield from tetramethylsilane (TMS) and are referenced to the carbon resonances of the solvent residual peak (DMSO-d_6 = δ 39.6 ppm). NMR data are presented as follows: chemical shift (δ ppm), multiplicity (s = singlet, d = doublet, t = triplet, q = quartet, m = multiplet, br = broad), coupling constant in Hertz (Hz), integration. Mass spectra were recorded on the Bruker MicrOTOF Q II. The experiments were performed triplicate, and all data were obtained based on the average values.

Growth Medium. Terrific Broth media was prepared as follows. For 1 L Terrific Broth media, deionized H_2O was added with 11.8 g of peptone 140 (pancreatic digest of casein), 23.6 g of yeast extract autolyzed low sodium, 9.4 g of dipotassium hydrogen phosphate, 2.2 g of potassium dihydrogen phosphate, 4 ml of glycerol and supplemented glucose (0.2 % w/v). Terrific Broth agar plates were prepared by adding 15 g agar to 1 L Terrific Broth media with ampicillin antibiotic selection and hemin (35 μg/mL). To media and plates was added ampicillin to a final concentration of 100 mg/L.

Cloning and mutagenesis.

Plasmid pET20b was used as a cloning and expression vector for all constructs described in this study. Site-saturation mutagenesis for amino acid residue 29 and CAST libraries were performed using primers containing degenerate codons (NNK; Table S4), PCR was performed using Q5® High-Fidelity DNA Polymerase (NEB) and the resulting PCR products were digested with DpnI (NEB). Then gel was purified (Omega), repaired (Vazyme) and the product was directly transformed to *E. coli* strain RP523 by incubating at 42°C. Following transformation, cells were recovered for 45 min at 37°C in Luria-Bertani (LB) medium, aliquots were plated on LB agar plates supplemented with 100 μg/mL ampicillin (LB-Amp plates), and plates were incubated at 37°C overnight. For Site-saturation mutagenesis, 80 colonies were picked to be cultured and screened.

For CAST libraries, we picked a library of 800-1000 colonies for high-throughput screening.

High-throughput UV analysis for library screening in 96-well plate format.

Single colonies of *E. coli* RP523 cells transformed with site-saturation or CAST libraries (or cells transformed with plasmid encoding the corresponding parent variant) were picked with sterile toothpicks and cultured in 96-deep-well plates in TB-Amp medium (1000 μL/well) at 37 °C, 250 rpm, overnight. In a fresh 96-deep-well plate, TB-Amp medium (950 μL/well) was inoculated with the pre-cultures (50 μL/well) and incubated at 37 °C, 250 rpm, for 12 h. The cells were induced by IPTG (0.3 mM) and metalloporphyrin solution (35 μg/mL) was added meanwhile. Expression was conducted at 16 °C, 200 rpm for 30 h. The cells were pelleted (3,000×g, 5 min), the supernatant was discarded, and the cell pellets were resuspended in reaction buffer (phosphate buffer (pH 7.4), 180 μL/well. Then stocks of **1a** and **2a** (final concentration 50 mM, 10 μL/well, in MeCN) and solution of K_2CO_3 (10 μL/well, in ddH_2O) were added. Following substrate addition, the plates were sealed with silicone covers and shaken at 500 rpm for 16 h, room temperature. After incubation, the seal was removed and reactions were worked up for high-throughput UV analysis. For high-throughput UV analysis, the plates were centrifuged (4,000×g, 10 min), and the cleared supernatant was filtered through an AcroPrep 96-well filter plate (0.2 μm cutoff) into a shallow-well plate for high-throughput UV analysis.

Small-scale reactions using whole cells.

Single colonies of *E. coli* RP523 cells transformed with plasmid encoding VHb variants were grown overnight in 3 mL TB-Amp medium at 37 °C and 200 rpm. 2 mL of the pre-cultures were used to inoculate 48 mL of TB-Amp medium and were incubated at 37 °C, 180 rpm for 3.5 h, typically reaching an OD_{600}=1.5. Cultures were then induced by IPTG in an anaerobic environment with the addition of metalloporphyrin. Expression was conducted at 25 °C, 110 rpm, for 30 h. Cultures were then centrifuged (3000×g, 3 min, 4 °C), and the pellets were resuspended to OD_{600}=30 in phosphate buffer (pH 7.4). The protein concentration in the whole-cell suspension was determined to 5 μM by lysis of an aliquot, and the CO binding assay as described earlier [1-2].

Small-scale biocatalytic reactions were set up in 1.5 mL EP tubes with 400 μL reaction volume. Typically, 360 μL of VHb-expressing cells at OD_{600}=30 in phosphate buffer were added to the tubes. Subsequently, the tubes were put on a shaker (300 rpm), and 20 μL of reagent **1a** and **2a** stock solution (final concentration 50 mM, dissolved in MeCN) and 20 μL of K_2CO_3 stock solution (final concentration 30 mM, dissolved in ddH_2O) were added. Following the addition of substrates, the tubes were shaken at room temperature, 500 rpm for 16 h. For sample work-up for HPLC analysis the reactions were quenched by adding extractive solvent (*n*-hexane: ethyl acetate=3:2, 200 μL/tube) supplemented with internal standard. The sample solutions were incubated for 30 min, transferred to 1.5 mL tubes and centrifuged at 13,000×g for 10 minutes. The cleared supernatant was transferred to clean 2 mL vials and analyzed by HPLC.

Small-scale reactions using cell lysates

Cells were lysed by sonication of 5–10 mL resuspended whole cells in 10 mM phosphate buffer (pH 7.4) on ice for 15 minutes at 40% amplitude (2 second on, 3 second off) using a sonicator and a 1/8-inch tip. The sonicated cell mixture was clarified via centrifugation at 4 °C and 15,000 g for 20 min. The lysate contained the expressed enzymes, and it was used for reactions and protein concentration

determination.

Protein expression and purification.

Single colonies were used to inoculate 5 mL of TB-Amp medium, followed by incubation at 37°C with shaking (180 rpm) for 10 to 15 hours. For expression of VHb, the overnight cultures were transferred to 1 L TB-Amp medium, followed by incubation at 37°C with shaking (180 rpm). At an OD_{600} of 1.5, cells were induced by IPTG in an anaerobic environment with the addition of metalloporphyrin and incubated at 16°C with shaking (110 rpm) for 30 hours. Cell cultures were harvested by centrifugation at 5000 rpm. The overall pelleted bacteria were dissolved in 50 mL of 20 mM phosphate buffer (pH 7.4). After sonication for 30 min on ice, the cell lysates were centrifuged at 12,000 rpm for 30 min. For purification, the lysate was transferred to a Ni-NTA column equilibrated with Ni-NTA Lysis Buffer. The resin was washed with 50 mL of Ni-NTA Lysis Buffer and then 50 mL of Ni-NTA Wash Buffer (20 mM phosphate buffer, 20 mM imidazole, pH = 7.4). Proteins were eluted with Ni-NTA Elution Buffer (20 mM phosphate buffer, 250 mM imidazole, pH = 7.4). After elution from the Ni-NTA column, the protein was loaded into a 5ml G-25 desalting column to remove imidazole. The concentration of the protein was tested by NanoDrop.

Molecular Docking Analysis.

The initial structure of VHB was taken from PDB code of 3tm3, structures of 1SM-VHb^{SD-Co} and 6SM-VHb^{SD-Co} was obtained by SWISS-MODEL[3] using homology modeling strategy. Intermediate **4** was docked into the active site of VHb^{SD-Co}, 1SM-VHb^{SD-Co} and 6SM-VHb^{SD-Co} using AutoDock Vina tool in Chimera respectively [4, 5]. The interactions between 6SM-VHb^{SD-Co} and intermediate **4** were showed in 2D diagram using Discovery Studio Visualizer 4.0[6].

Synthetic Procedures.

Preparative scale procedure for enzymatic synthesis of products **3** (**Procedure A**):

To a 5 L Erlenmeyer flask containing 2.5 L of a suspension of 6SM-VHb^{SD-Co} expressing cells (*E. coli* RP523, OD_{600}=200). Following expression, the cultures were centrifuged at 4 °C and 4,000 g for 30 min. The cell pellets were then resuspended in phosphate buffer (10 mM, pH 7.4, 25 mL per pellet), and the cell suspensions were combined. Then the lysed by sonication of 25 mL resuspended whole cells in 10 mM phosphate buffer (pH 7.4) on ice for 1.5 minutes at 40% amplitude (2 second on, 3 second off) using a sonicator and a 1/8-inch tip. The suspension of 6SM-VHb^{SD-Co} was concentrated to 3 mg/ml, and then 800 ul was added to the mixture of 100 ul amidines and isothiocyanate stock solution (5 M, 0.5 mmol in MeCN) and 100 ul K_2CO_3 solution (3 M, 0.3 mmol in ddH_2O). The flask was sealed with a rubber septum and inflated with O_2 for 15 min. The reaction mixture was stirred 16 h at room temperature under positive oxygen pressure. The desired product was extracted with CH_2Cl_2 (3 x 5 mL) and washed with water and brine. The organic layers were combined and dried over sodium sulfate, evaporated under reduced pressure, and the residue was purified by column chromatography (hexanes/EtOAc, 20:1 to 2:1) to afford the product. The yields of products were determined by HPLC.

Supporting Experimental Tables and Figures

Table S1. Optimization of Reaction Conditions [a]

Entry	Protein	Solvent	Concentration of K_2CO_3	Time	Yield (%) [b]	TON [c]
1	VHb^{SD-Co} (0.05 mol%)	PBS	30 mM	12 h	62	1240
2	VHb^{SD-Co} (0.05 mol%)	MeCN	30 mM	12 h	25	500
3	VHb^{SD-Co} (0.05 mol%)	MeOH	30 mM	12 h	6	120
4	VHb^{SD-Co} (0.05 mol%)	DMSO	30 mM	12 h	18	360
5	VHb^{SD-Co} (0.05 mol%)	PBS (5% MeCN)	30 mM	12 h	69	1380
6	VHb^{SD-Co} (0.05 mol%)	PBS (5% MeCN)	10 mM	12 h	48	960
7	VHb^{SD-Co} (0.05 mol%)	PBS (5% MeCN)	20 mM	12 h	59	1180
8	VHb^{SD-Co} (0.05 mol%)	PBS (5% MeCN)	40 mM	12 h	63	1260
9	VHb^{SD-Co} (0.05 mol%)	PBS (5% MeCN)	50 mM	12 h	49	980
10	VHb^{SD-Co} (0.05 mol%)	PBS (5% MeCN)	30 mM	10 h	56	1120
11	VHb^{SD-Co} (0.05 mol%)	PBS (5% MeCN)	30 mM	14 h	70	1400
12	VHb^{SD-Co} (0.05 mol%)	PBS (5% MeCN)	30 mM	16 h	71	1420
13	VHb^{SD-Co} (0.05 mol%)	PBS (5% MeCN)	30 mM	18 h	71	1420
14	VHb^{SD-Co} (0.05 mol%)	PBS (5% MeCN)	30 mM	24 h	71	1420
15	VHb^{SD-Co} (0.01 mol%)	PBS (5% MeCN)	30 mM	16 h	19	380
16	VHb^{SD-Co} (0.03 mol%)	PBS (5% MeCN)	30 mM	16 h	54	1080
17	VHb^{SD-Co} (0.07 mol%)	PBS (5% MeCN)	30 mM	16 h	71	1420

a. Reaction condition: benzamidines (**1a**, 50 mM), phenyl isothiocyanate (**2a**, 50 mM), VHb^{SD-Co} (metalloporphyrin containing 0.05 mol%), O_2, solvent. b. Determined by high-performance liquid chromatography (HPLC).

Table S2. Evaluation of Previously Reported VHb Variants [a]

Entry	Protein	Yield (%) [b]	TON
1	VHb^{Co} (Q53H)	67	1340
2	VHb^{Co} (P54C)	58	1160
3	VHb^{Co} (Q53H, P54C)	63	1260
4	VHb (H85Y)	35	780
5	VHb^{Co} (H85Y)	7	140
6	VHb (H85S)	37	820
7	VHb^{Co} (H85S)	16	320
8	VHb (H85C)	19	380
9	VHb^{Co} (H85C)	12	240
10	VHb (H85A)	-	-

11	VHb (H85V)	-	-

a. Reaction condition: benzamidines (**1a**, 50 mM), phenyl isothiocyanate (**2a**, 50 mM), VHb-based catalysts (metalloporphyrin containing 0.05 mol%), K_2CO_3 30 mM, O_2, PBS buffer (10 mM, 5% MeCN), stirred at rt for 16 h. b. Determined by HPLC.

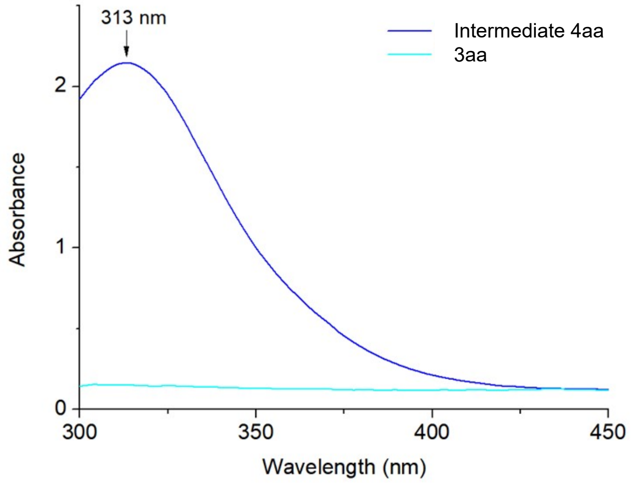

Figure S1. UV absorbance of intermediate **4aa** and product **3aa**

In the second round of CAST screening, we picked approximately 1000 colonies for whole-cell screening. As a positive control, the parent mutant (1SM-VHb$^{SD\text{-}Co}$) was also included in the screening and cultured under identical conditions. The whole-cell catalyst containing the original mutant 1SM-VHb$^{SD\text{-}Co}$, on average, catalyzed the formation of product **3aa** with 81 % yield. Therefore, only the colonies that produced **3aa** with yields higher than or equal to 81% were selected and sequenced. Out of the ten 96-well plates, we obtained 48 mutants that led to improved selectivity. Among these 48 "hits", 8 were the parent mutants, the top 10 "hits" were listed in **Table S3** and the mutant that afforded the highest yield contained 6 mutations (VHb$^{SD\text{-}Co}$ Y29G-F43P-Q53P-P54G-K55L-L57A defined as 6SM-VHb$^{SD\text{-}Co}$).

Table S3. Screening of CAST library of F43, Q53, P54, K55 and L57 based on 1SM-VHb$^{SD\text{-}Co}$

	Mutations	Yield of **3aa** (TON)
Hit 1	Y29G+F43P,Q53P,P54G,K55L,L57A	92.8% (1856)
Hit 2	Y29G+Q53R,P54N,K55L,L57Q	90.2% (1804)
Hit 3	Y29G+Q53G,P54L,K55D,L57R	89.5% (1790)
Hit 4	Y29G+Q53P,L57D	89.3% (1786)
Hit 5	Y29G+Q53G,P54L,K55G,L57R	88.9% (1778)
Hit 6	Y29G+F43V,Q53W,P54H,K55R,L57D	88.0% (1760)
Hit 7	Y29G+Q53P	87.4% (1748)
Hit 8	Y29G+F43V,P54G,L57P	86.2% (1724)
Hit 9	Y29G+F43A,K55T	85.8% (1716)
Hit 10	Y29G+F43A,Q53G,P54L,K55S,L57P	85.3% (1706)

Table S4. Comparison of mutants with Co(ppIX) and Fe(ppIX) cofactor along the evolutionary trajectory

Entry	Protein	Yield (%) ^{b}	TON ^{c}
1	VHb$^{SD\text{-}Co}$	71	1420
2	VHb^{SD}	45	900
3	1SM-VHb$^{SD\text{-}Co}$	81	1620
4	1SM-VHb^{SD}	53	1060
5	6SM-VHb$^{SD\text{-}Co}$	93	1860
6	6SM-VHb^{SD}	64	1280

a. Reaction condition: benzamidines (**1a**, 50 mM), phenyl isothiocyanate (**2a**, 50 mM), VHb$^{SD\text{-}Co}$ (metalloporphyrin containing 0.05 mol%), K_2CO_3 30 mM, O_2, PBS buffer (10 mM, 5% MeCN), stirred at rt for 16 h. b. Determined by HPLC.

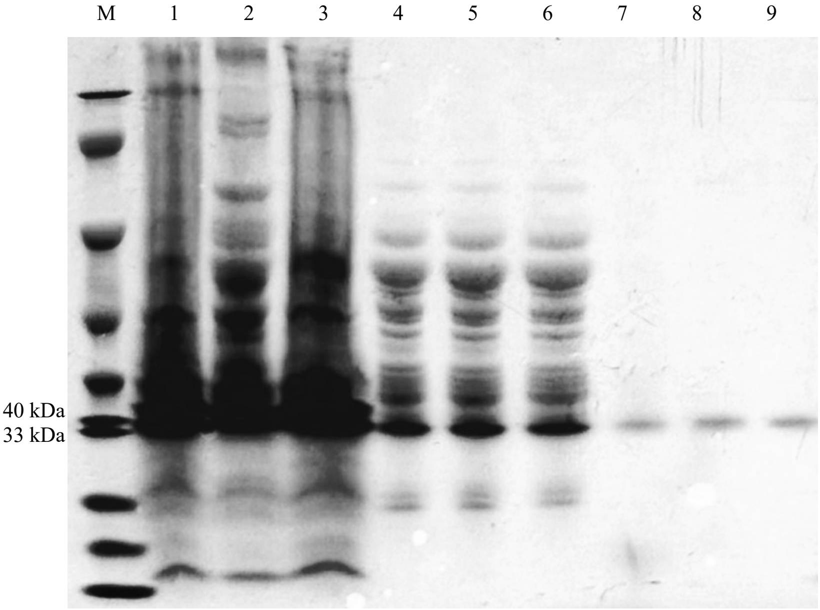

Figure S2. SDS-PAGE analysis of VHb$^{SD\text{-}Co}$. M: Marker; 1: Cell pellets of VHb$^{SD\text{-}Co}$; 2: Cell pellets of 1SM-VHb$^{SD\text{-}Co}$; 3: Cell pellets of 6SM-VHb$^{SD\text{-}Co}$; 4: Supernatant of VHb$^{SD\text{-}Co}$ after cell lysis and centrifugation; 5: Supernatant of 1SM-VHb$^{SD\text{-}Co}$ after cell lysis and centrifugation; 6: Supernatant of 6SM-VHb$^{SD\text{-}Co}$ after cell lysis and centrifugation; 7: Purified VHb$^{SD\text{-}Co}$; 8: Purified 1SM-VHb$^{SD\text{-}Co}$; 9: Purified 6SM-VHb$^{SD\text{-}Co}$.

Detecti[illegible]ion mixture

a) 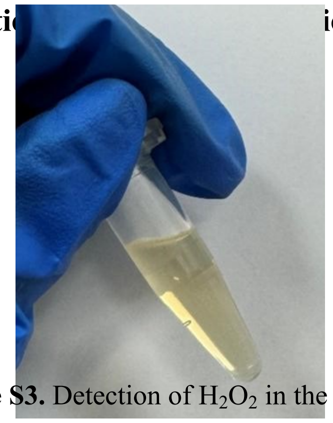 b) 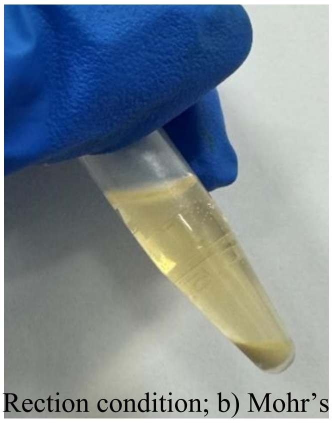

Figure S3. Detection of H_2O_2 in the reaction mixture. a) Rection condition; b) Mohr's salt added.

During the detection of H_2O_2 in this reaction, we chose the reaction condition: O_2 used as oxidant under aerobic conditions, VHb^{Co} as catalyst (Co(ppIX) containing 0.05 mol%), **1a** (50 mM), **2a** (50 mM), K_2CO_3 (30 mM) in phosphate-buffered solution (PBS) (5% MeCN) for 16 h. After 12 h of reaction, the reaction system was centrifuged at 12,000 rpm for 1 min, a 100 μL solution of Mohr's salt (10 mg in 100 μL H_2O) was added to the reaction mixture. Then, a rapid formation of $Fe(OH)_3$ floc was observed. The floc was observed due to the rapid oxidation of Fe(II) to Fe(III) by H_2O_2 in the reaction mixture.

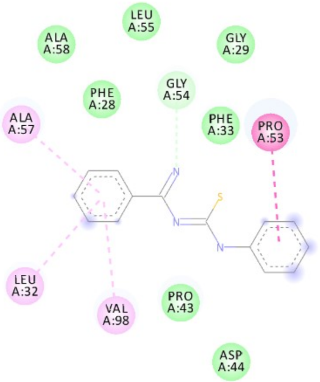

Figure S4. 2D diagram showing the interactions of the intermediate **4** and the residues in the cavity of $6SM\text{-}VHb^{SD\text{-}Co}$. Van der Waals interactions (green); carbon hydrogen bond (light green); amide-pi stacked (pink); pi-alkyl (light pink).

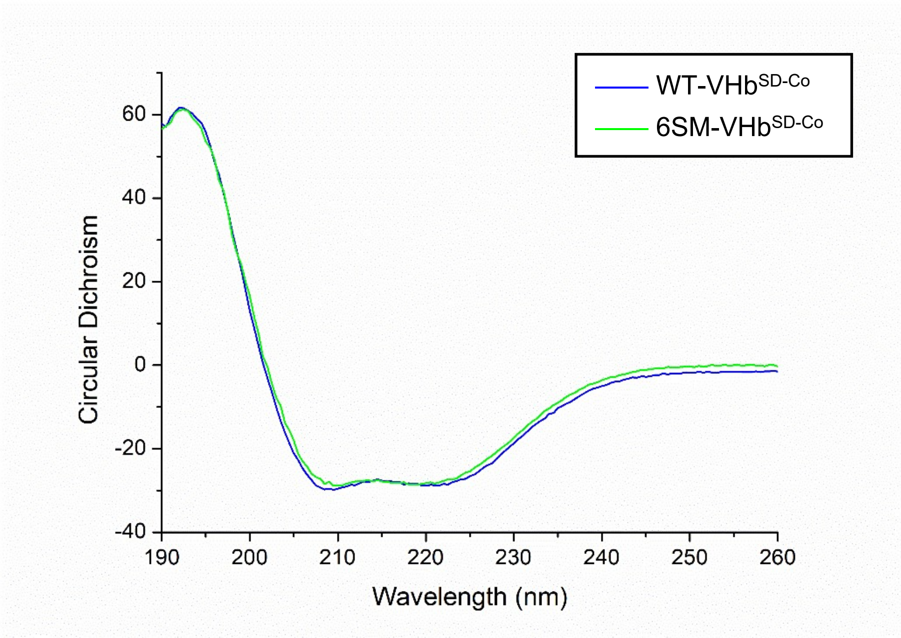

Figure S5. CD spectra of WT-VHb^{SD-Co} and 6SM-VHb^{SD-Co}.

Sequence of primers and variants

Table S5. Primers of PCR reactions

Primer	Sequence
Lpp-OmpA-VHb-F	GGTTACCCATATGATGAAAGCGACCAAACT
Lpp-OmpA-VHb-R	CCGCCGGAATTCTTAATGATGATGATGATGAT
29-F	CCACGACTTTTNNKAAAAACTTGTTTGCCAAAACA
29-R	CAAACAAGTTTTTMNNAAAAGTCGTGGTAATGGTAACGC
5SSM-F	GGGTCGCCAAGAATCTTTGGAGNNKNNKNNKGCTNNKGCGA TGACGGTATT
5SSM-R	CCAAAGATTCTTGGCGACCCATATCMNNCAAAGGACGTACTT CAGGGTGTTT

Nucleotide Sequence of 6SM-VHb$^{SD\text{-}Co}$

ATGAAAGCGACCAAACTGGTGCTGGGCGCGGTGATTCTGGGCAGCACCCTGCTGGCG
GGCTGCAGCAGCAACGCGAAAATTGATCAGAACAACAACGGCCCGACCCATGAAAA
CCAGCTGGGCGCGGGCGCGTTTGGCGGCTATCAGGTGAACCCGTATGTGGGCTTTGA
AATGGGCTATGATTGGCTGGGCCGCATGCCGTATAAAGGCAGCGTGGAAAACGGCGC
GTATAAAGCGCAGGGCGTGCAGCTGACCGCGAAACTGGGCTATCCGATTACCGATGA
TCTGGATATTTATACCCGCCTGGGCGGCATGGTGTGGCGCGCGGATACCAAAAGCAA
CGTGTATGGCAAAAACCATGATACCGGCGTGAGCCCGGTGTTTGCGGGCGGCGTGGA
ATATGCGATTACCCCGGAAATTGCGACCGGATCCGGCGGAGGCGGAGGCATGTTAGA
CCAGCAAACCATTAACATCATCAAAGCCACTGTTCCTGTATTGAAGGAGCATGGCGTT
ACCATTACCACGACTTTTGGCAAAAACTTGTTTGCCAAACACCCTGAAGTACGTCCTT
TGCCGGATATGGGTCGCCAAGAATCTTTGGAGCCTGGGCTGGCTGCGGCGATGACGG
TATTGGCGGCAGCGCAAAACATTGAAAATTTGCCAGCTATTTTGCCTGCGGTCAAAA
AAATTGCAGTCAAACATTGTCAAGCAGGCGTGGCAGCAGCGCATTATCCGATTGTCG
GTCAAGAATTGTTGGGTGCGATTAAAGAAGTATTGGGCGATGCCGCAACCGATGACA
TTTTGGACGCGTGGGGCAAGGCTTATGGCGTGATTGCAGATGTGTTTATTCAAGTGGA
AGCAGATTTGTACGCTCAAGCGGTTGAACATCATCATCATCATCATTAA

Green: Lpp; yellow: OpmA; grey: 5xGly link; blue: 6SM-VHb (red: mutations)

Amino Acid Sequence of 6SM-VHb$^{SD\text{-}Co}$

MKATKLVLGAVILGSTLLAGCSSNAKIDQNNNGPTHENQLGAGAFGGYQVNPYVGFEM
GYDWLGRMPYKGSVENGAYKAQGVQLTAKLGYPITDDLDIYTRLGGMVWRADTKSNV
YGKNHDTGVSPVFAGGVEYAITPEIATGSGGGGGMLDQQTINIIKATVPVLKEHGVTITTT
FGKNLFAKHPEVRPLPDMGRQESLEPGLAAAMTVLAAAQNIENLPAILPAVKKIAVKHCQ
AGVAAAHYPIVGQELLGAIKEVLGDAATDDILDAWGKAYGVIADVFIQVEADLYAQAVE
HHHHHH*

Green: Lpp; yellow: OpmA; grey: 5xGly link; blue: 6SM-VHb (red: mutations)

NMR Data of Products

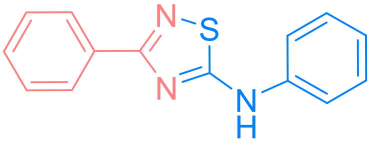

***N*,3-diphenyl-1,2,4-thiadiazol-5-amine (3aa)** [7]

Following the standard synthesis procedure, white solid. ^{1}H NMR (400 MHz, Chloroform-*d*) δ 11.06 (s, 1H), 8.21 – 8.17 (m, 2H), 7.68 – 7.64 (m, 2H), 7.54 – 7.50 (m, 3H), 7.47 – 7.42 (m, 2H), 7.11 (tt, *J* = 7.2, 1.2 Hz, 1H).

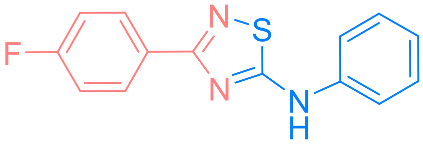

3-(4-fluorophenyl)-*N*-phenyl-1,2,4-thiadiazol-5-amine (3ba) [12]

Following the standard synthesis procedure, white solid. ^{1}H NMR (400 MHz, DMSO-d_6) δ 11.07 (s, 1H), 8.27 – 8.21 (m, 2H), 7.67 (d, *J* = 8.0 Hz, 2H), 7.45 (dd, *J* = 8.4, 7.2 Hz, 2H), 7.37 (t, *J* = 8.8 Hz, 2H), 7.12 (t, *J* = 7.2 Hz, 1H).

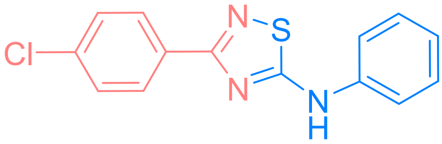

3-(4-chlorophenyl)-*N*-phenyl-1,2,4-thiadiazol-5-amine (3ca) [12]

Following the standard synthesis procedure, white solid. ^{1}H NMR (400 MHz, DMSO-d_6) δ 11.10 (s, 1H), 8.23 – 8.18 (m, 2H), 7.69 – 7.65 (m, 2H), 7.63 – 7.59 (m, 2H), 7.48 – 7.43 (m, 2H), 7.13 (tt, *J* = 7.2, 1.2 Hz, 1H).

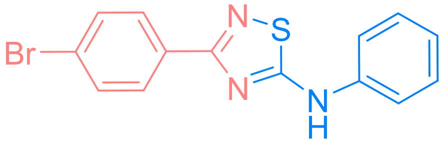

3-(4-bromophenyl)-*N*-phenyl-1,2,4-thiadiazol-5-amine (3da) [12]

Following the standard synthesis procedure, white solid. ^{1}H NMR (400 MHz, DMSO-d_6) δ 11.10 (s, 1H), 8.16 – 8.10 (m, 2H), 7.80 – 7.72 (m, 2H), 7.71 – 7.64 (m, 2H), 7.50 – 7.42 (m, 2H), 7.13 (tt, *J* = 7.6, 1.2 Hz, 1H).

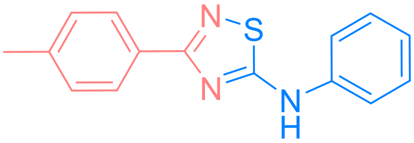

***N*-phenyl-3-(p-tolyl)-1,2,4-thiadiazol-5-amine (3ea)** [9]

Following the standard synthesis procedure, white solid. ^{1}H NMR (400 MHz, DMSO-d_6) δ 11.06 (s,

1H), 8.11 – 8.07 (m, 2H), 7.69 – 7.64 (m, 2H), 7.48 – 7.43 (m, 2H), 7.34 (d, *J* = 8.0 Hz, 2H), 7.12 (tt, *J* = 7.2, 1.2 Hz, 1H), 2.39 (s, 3H).

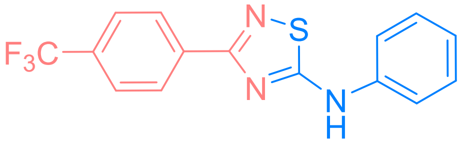

***N*-phenyl-3-(4-(trifluoromethyl)phenyl)-1,2,4-thiadiazol-5-amine (3fa)** [14]

Following the standard synthesis procedure, white solid. ^{1}H NMR (400 MHz, DMSO-d_6) δ 11.16 (s, 1H), 8.39 (d, *J* = 8.0 Hz, 2H), 7.92 (d, *J* = 8.0 Hz, 2H), 7.68 (d, *J* = 8.0 Hz, 2H), 7.46 (dd, *J* = 8.4, 7.2 Hz, 2H), 7.14 (t, *J* = 7.2 Hz, 1H).

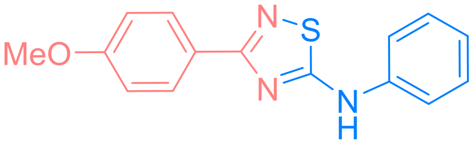

3-(4-methoxyphenyl)-*N*-phenyl-1,2,4-thiadiazol-5-amine (3ga) [12]

Following the standard synthesis procedure, white solid. ^{1}H NMR (400 MHz, DMSO-d_6) δ 11.02 (s, 1H), 8.16 – 8.12 (m, 2H), 7.69 – 7.64 (m, 2H), 7.47 – 7.41 (m, 2H), 7.13 – 7.05 (m, 3H), 3.85 (s, 3H).

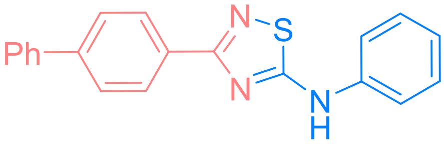

3-([1,1'-biphenyl]-4-yl)-*N*-phenyl-1,2,4-thiadiazol-5-amine (3ha)

Following the standard synthesis procedure, white solid. ^{1}H NMR (400 MHz, DMSO-d_6) δ 11.09 (s, 1H), 8.31 – 8.26 (m, 2H), 7.87 – 7.83 (m, 2H), 7.79 – 7.75 (m, 2H), 7.72 – 7.68 (m, 2H), 7.52 (dd, *J* = 8.4, 7.2 Hz, 2H), 7.49 – 7.40 (m, 3H), 7.15 – 7.10 (m, 1H). ^{13}C NMR (101 MHz, DMSO-d_6) δ 179.60, 168.78, 142.16, 140.34, 139.82, 132.24, 129.96, 129.57, 128.70, 128.44, 127.51, 127.23, 123.49, 118.20. HRMS (ESI): *m/z* = (M + H$^+$) calcd for $C_{20}H_{15}N_3S$: 329.0987, found: 329.0985.

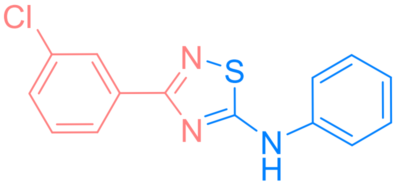

3-(3-chlorophenyl)-*N*-phenyl-1,2,4-thiadiazol-5-amine (3ia)

Following the standard synthesis procedure, white solid. ^{1}H NMR (400 MHz, DMSO-d_6) δ 11.13 (s, 1H), 8.15 (hept, *J* = 1.6 Hz, 1H), 7.68 – 7.64 (m, 1H), 7.59 (dd, *J* = 4.8, 2.4 Hz, 1H), 7.49 – 7.44 (m, 1H), 7.16 – 7.10 (m, 1H). ^{13}C NMR (101 MHz, DMSO-d_6) δ 179.89, 167.54, 140.15, 135.00, 133.99, 131.40, 130.55, 130.00, 127.50, 126.69, 123.69, 118.30. HRMS (ESI): *m/z* = (M + H$^+$) calcd for $C_{14}H_{10}ClN_3S$: 287.0284, found: 287.0283.

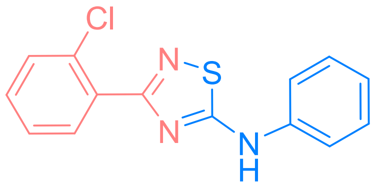

3-(2-chlorophenyl)-*N*-phenyl-1,2,4-thiadiazol-5-amine (3ja)

Following the standard synthesis procedure, white solid. ^{1}H NMR (400 MHz, DMSO-d_6) δ 11.11 (s, 1H), 7.91 (dd, *J* = 7.2, 2.4 Hz, 1H), 7.67 – 7.61 (m, 3H), 7.51 (pd, *J* = 7.2, 1.6 Hz, 2H), 7.45 – 7.40 (m, 2H), 7.10 (t, *J* = 7.2 Hz, 1H). ^{13}C NMR (101 MHz, DMSO-d_6) δ 179.15, 167.52, 140.18, 132.75, 132.28, 132.22, 131.66, 131.04, 129.92, 127.82, 123.57, 118.20. HRMS (ESI): *m/z* = (M + H$^+$) calcd for $C_{14}H_{10}ClN_3S$: 287.0284, found: 287.0285.

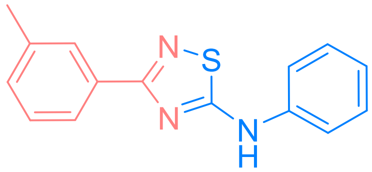

***N*-phenyl-3-(m-tolyl)-1,2,4-thiadiazol-5-amine (3ka)** [14]

Following the standard synthesis procedure, white solid. ^{1}H NMR (400 MHz, DMSO-d_6) δ 11.08 (s, 1H), 8.03 – 7.98 (m, 2H), 7.69 – 7.64 (m, 2H), 7.48 – 7.40 (m, 3H), 7.33 (ddt, *J* = 7.6, 2.0, 1.2 Hz, 1H), 7.12 (tt, *J* = 7.2, 1.2 Hz, 1H), 2.42 (s, 3H).

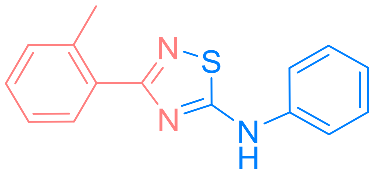

***N*-phenyl-3-(o-tolyl)-1,2,4-thiadiazol-5-amine (3la)**

Following the standard synthesis procedure, white solid. ^{1}H NMR (400 MHz, DMSO-d_6) δ 11.04 (s, 1H), 8.03 – 7.92 (m, 1H), 7.68 – 7.63 (m, 2H), 7.45 – 7.41 (m, 2H), 7.40 – 7.32 (m, 3H), 7.10 (tt, *J* = 7.2, 1.2 Hz, 1H), 2.64 (s, 3H). ^{13}C NMR (101 MHz, DMSO-d_6) δ 178.70, 170.22, 140.38, 137.43, 132.94, 131.83, 130.76, 130.00, 129.90, 126.42, 123.36, 118.10, 22.26. HRMS (ESI): *m/z* = (M + H$^+$) calcd for $C_{15}H_{13}N_3S$: 267.0830, found: 267.0829.

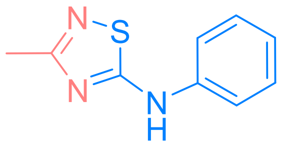

3-methyl-*N*-phenyl-1,2,4-thiadiazol-5-amine (3ma) [7]

Following the standard synthesis procedure, white solid. ^{1}H NMR (400 MHz, DMSO-d_6) δ 10.89 (s, 1H), 7.58 – 7.54 (m, 2H), 7.41 – 7.37 (m, 2H), 7.06 (tt, *J* = 7.2, 1.2 Hz, 1H), 2.41 (s, 3H).

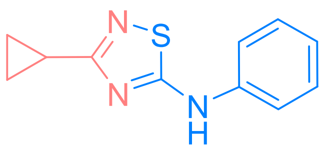

3-cyclopropyl-*N*-phenyl-1,2,4-thiadiazol-5-amine (3na) [15]

Following the standard synthesis procedure, white solid. ^{1}H NMR (400 MHz, DMSO-d_6) δ 10.89 (s, 1H), 7.51 – 7.47 (m, 2H), 7.42 – 7.37 (m, 2H), 7.07 (tt, J = 7.2, 1.2 Hz, 1H), 2.14 – 2.07 (m, 1H), 1.01 – 0.97 (m, 4H).

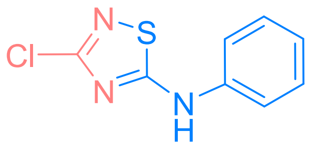

3-chloro-*N*-phenyl-1,2,4-thiadiazol-5-amine (3oa)

Following the standard synthesis procedure, white solid. ^{1}H NMR (400 MHz, DMSO-d_6) δ 10.49 (s, 1H), 7.84 – 7.80 (m, 3H), 7.40 – 7.34 (m, 2H), 7.06 – 7.02 (m, 1H). ^{13}C NMR (101 MHz, DMSO-d_6) δ 183.73, 167.79, 140.43, 129.54, 124.37, 120.01, 77.42, 77.10, 76.79. HRMS (ESI): m/z = (M + H$^+$) calcd for $C_8H_6ClN_3S$: 210.9971, found: 210.9970.

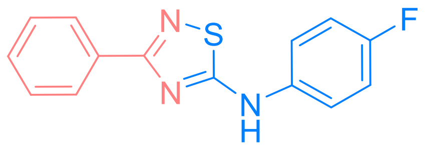

***N*-(4-fluorophenyl)-3-phenyl-1,2,4-thiadiazol-5-amine (3ab)** [9]

Following the standard synthesis procedure, white solid. ^{1}H NMR (400 MHz, DMSO-d_6) δ 11.06 (s, 1H), 8.23 – 8.16 (m, 2H), 7.72 (dd, J = 8.8, 4.8 Hz, 2H), 7.53 (qd, J = 4.8, 1.6 Hz, 3H), 7.30 (t, J = 8.8 Hz, 2H).

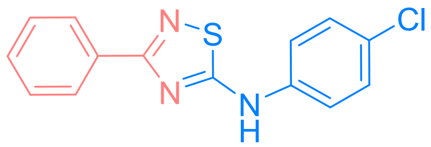

***N*-(4-chlorophenyl)-3-phenyl-1,2,4-thiadiazol-5-amine (3ac)** [8]

Following the standard synthesis procedure, white solid. ^{1}H NMR (400 MHz, DMSO-d_6) δ 11.17 (s, 1H), 8.23 – 8.18 (m, 2H), 7.77 – 7.71 (m, 2H), 7.56 – 7.48 (m, 5H).

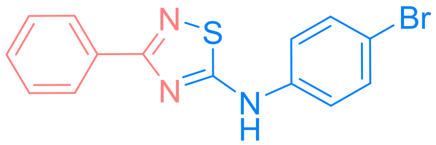

***N*-(4-bromophenyl)-3-phenyl-1,2,4-thiadiazol-5-amine (3ad)** [15]

Following the standard synthesis procedure, white solid. ^{1}H NMR (400 MHz, DMSO-d_6) δ 11.17 (s, 1H), 8.22 – 8.18 (m, 2H), 7.68 (d, J = 8.8 Hz, 2H), 7.62 (d, J = 8.8 Hz, 2H), 7.56 – 7.51 (m, 3H).

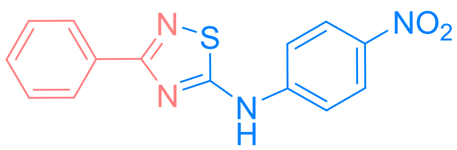

N-(4-nitrophenyl)-3-phenyl-1,2,4-thiadiazol-5-amine (3ae) [9]

Following the standard synthesis procedure, white solid. ^{1}H NMR (400 MHz, DMSO-d_6) δ 11.23 (s, 1H), 8.21 – 8.18 (m, 2H), 7.88 (t, J = 2.0 Hz, 1H), 7.61 – 7.53 (m, 4H), 7.47 (t, J = 8.0 Hz, 1H), 7.17 (ddd, J = 8.0, 2.0, 0.8 Hz, 1H).

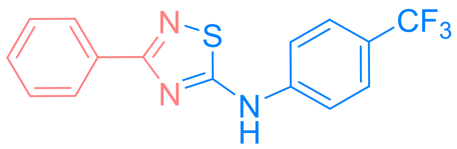

3-phenyl-N-(4-(trifluoromethyl)phenyl)-1,2,4-thiadiazol-5-amine (3af) [14]

Following the standard synthesis procedure, white solid. ^{1}H NMR (400 MHz, DMSO-d_6) δ 11.41 (s, 1H), 8.22 (dd, J = 7.2, 2.8 Hz, 2H), 7.91 (d, J = 8.4 Hz, 2H), 7.79 (d, J = 8.4 Hz, 2H), 7.53 (t, J = 3.6 Hz, 3H).

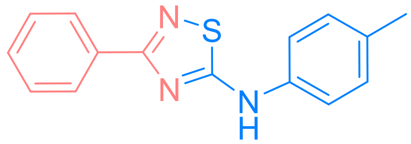

3-phenyl-N-(p-tolyl)-1,2,4-thiadiazol-5-amine (3ag) [8]

Following the standard synthesis procedure, white solid. ^{1}H NMR (400 MHz, DMSO-d_6) δ 10.98 (s, 1H), 8.21 – 8.17 (m, 2H), 7.55 – 7.50 (m, 5H), 7.25 (d, J = 8.0 Hz, 2H), 2.31 (s, 3H).

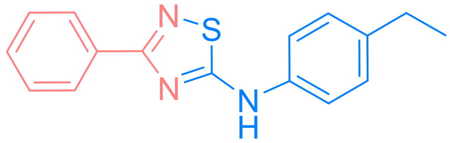

N-(4-ethylphenyl)-3-phenyl-1,2,4-thiadiazol-5-amine (3ah) [13]

Following the standard synthesis procedure, white solid. ^{1}H NMR (400 MHz, DMSO-d_6) δ 10.99 (s, 1H), 8.22 – 8.18 (m, 2H), 7.58 – 7.50 (m, 5H), 7.30 – 7.26 (m, 2H), 2.61 (q, J = 7.6 Hz, 2H), 1.20 (t, J = 7.6 Hz, 3H).

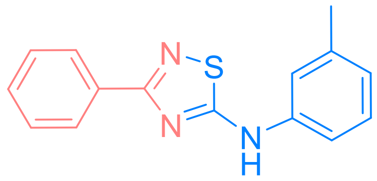

3-phenyl-N-(m-tolyl)-1,2,4-thiadiazol-5-amine (3ai) [8]

Following the standard synthesis procedure, white solid. ^{1}H NMR (400 MHz, DMSO-d_6) δ 11.02 (s, 1H), 8.24 – 8.17 (m, 2H), 7.52 (ddt, J = 12.0, 10.4, 3.6 Hz, 4H), 7.40 (s, 1H), 7.34 (t, J = 7.6 Hz, 1H), 6.95 (d, J = 7.6 Hz, 1H), 2.37 (s, 3H).

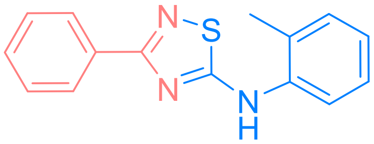

3-phenyl-*N*-(o-tolyl)-1,2,4-thiadiazol-5-amine (3aj) [8]

Following the standard synthesis procedure, white solid. ^{1}H NMR (400 MHz, DMSO-d_6) δ 10.26 (s, 1H), 8.14 (dd, J = 7.2, 2.4 Hz, 2H), 7.98 (d, J = 8.4 Hz, 1H), 7.51 (dt, J = 4.8, 2.8 Hz, 3H), 7.31 (d, J = 7.2 Hz, 2H), 7.17 – 7.12 (m, 1H), 2.33 (s, 3H).

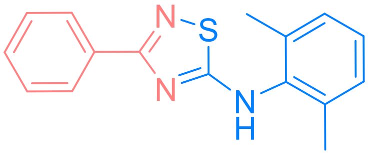

***N*-(2,6-dimethylphenyl)-3-phenyl-1,2,4-thiadiazol-5-amine (3ak)** [16]

Following the standard synthesis procedure, white solid. ^{1}H NMR (400 MHz, DMSO-d_6) δ 10.23 (s, 1H), 8.08 (dd, J = 6.8, 3.2 Hz, 2H), 7.48 (dd, J = 5.2, 2.0 Hz, 3H), 7.22 (s, 3H), 2.26 (s, 6H).

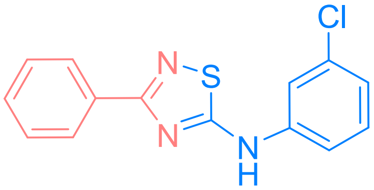

***N*-(3-chlorophenyl)-3-phenyl-1,2,4-thiadiazol-5-amine (3al)** [10]

Following the standard synthesis procedure, white solid. ^{1}H NMR (400 MHz, DMSO-d_6) δ 11.68 (s, 1H), 8.37 – 8.32 (m, 2H), 8.27 – 8.23 (m, 2H), 7.98 – 7.93 (m, 2H), 7.55 (dd, J = 5.2, 2.0 Hz, 3H).

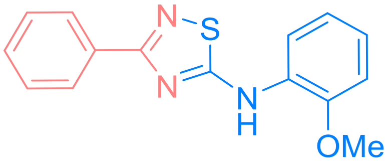

***N*-(2-methoxyphenyl)-3-phenyl-1,2,4-thiadiazol-5-amine (3am)** [9]

Following the standard synthesis procedure, white solid. ^{1}H NMR (400 MHz, DMSO-d_6) δ 10.48 (s, 1H), 8.55 – 8.50 (m, 1H), 8.23 – 8.18 (m, 2H), 7.58 – 7.47 (m, 3H), 7.14 – 7.06 (m, 3H), 3.92 (s, 3H).

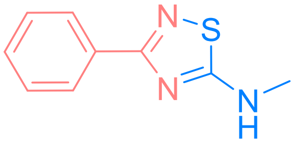

***N*-methyl-3-phenyl-1,2,4-thiadiazol-5-amine (3an)** [11]

Following the standard synthesis procedure, white solid. ^{1}H NMR (400 MHz, Chloroform-d) δ 8.47 (s, 1H), 8.11 – 8.07 (m, 1H), 7.45 (qd, J = 4.0, 1.6 Hz, 1H), 2.96 (d, J = 4.8 Hz, 2H).

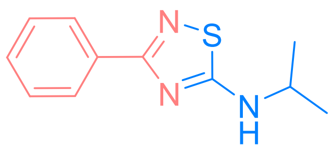

***N*-isopropyl-3-phenyl-1,2,4-thiadiazol-5-amine (3ao)** [11]

Following the standard synthesis procedure, white solid. ^{1}H NMR (400 MHz, Chloroform-*d*) δ 8.49 (s, 1H), 8.08 (dq, *J* = 8.0, 3.2, 2.8 Hz, 2H), 7.50 – 7.42 (m, 3H), 3.81 (s, 1H), 1.24 (d, *J* = 6.4 Hz, 6H).

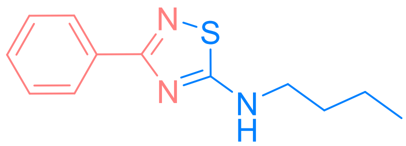

***N*-butyl-3-phenyl-1,2,4-thiadiazol-5-amine (3ap)** [17]

Following the standard synthesis procedure, white solid. ^{1}H NMR (400 MHz, DMSO-d_6) δ 8.57 (s, 1H), 8.12 – 8.08 (m, 2H), 7.47 (dd, *J* = 5.2, 2.0 Hz, 3H), 3.35 (s, 2H), 1.64 – 1.57 (m, 2H), 1.44 – 1.34 (m, 2H), 0.93 (t, *J* = 7.2 Hz, 3H).

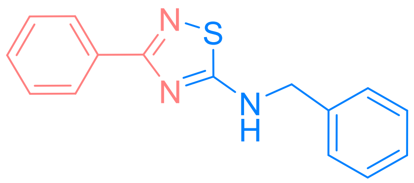

***N*-benzyl-3-phenyl-1,2,4-thiadiazol-5-amine (3aq)** [10]

Following the standard synthesis procedure, white solid. ^{1}H NMR (400 MHz, DMSO-d_6) δ 9.04 (s, 1H), 8.13 – 8.09 (m, 2H), 7.48 (dd, *J* = 5.2, 2.0 Hz, 3H), 7.44 – 7.35 (m, 4H), 7.33 – 7.28 (m, 1H), 4.62 (d, *J* = 5.6 Hz, 2H).

^{1}H NMR Spectra

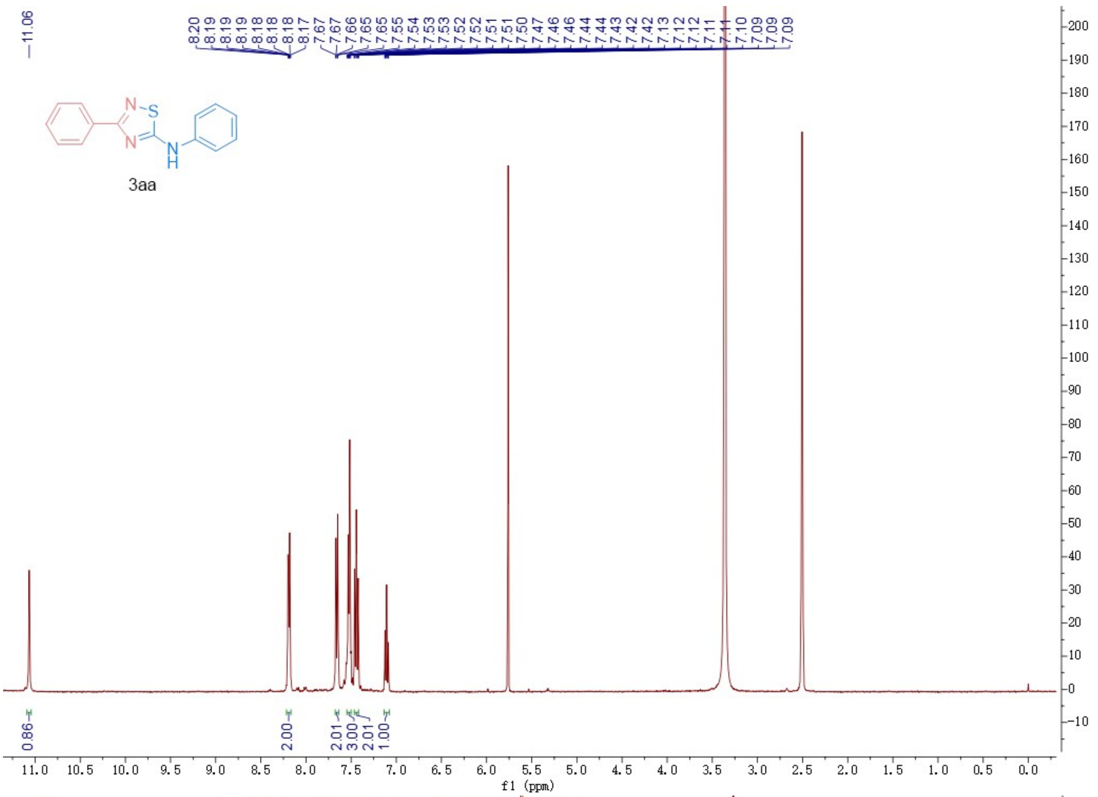

3aa

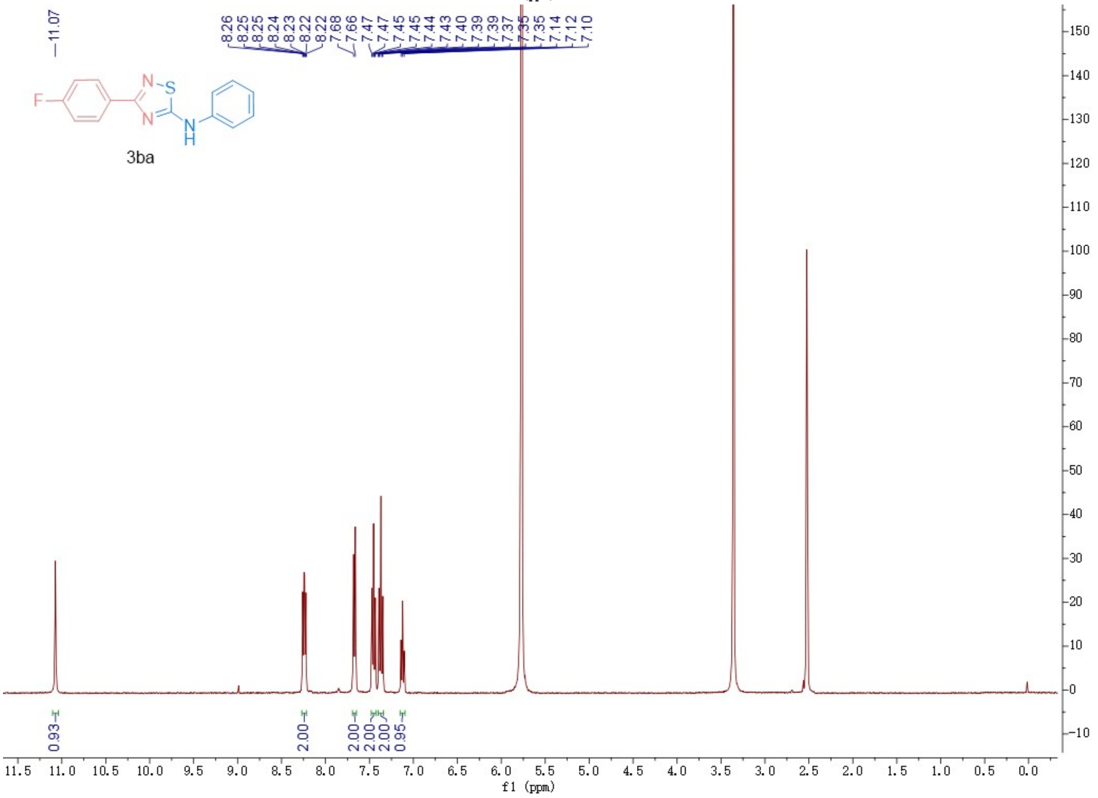

3ba

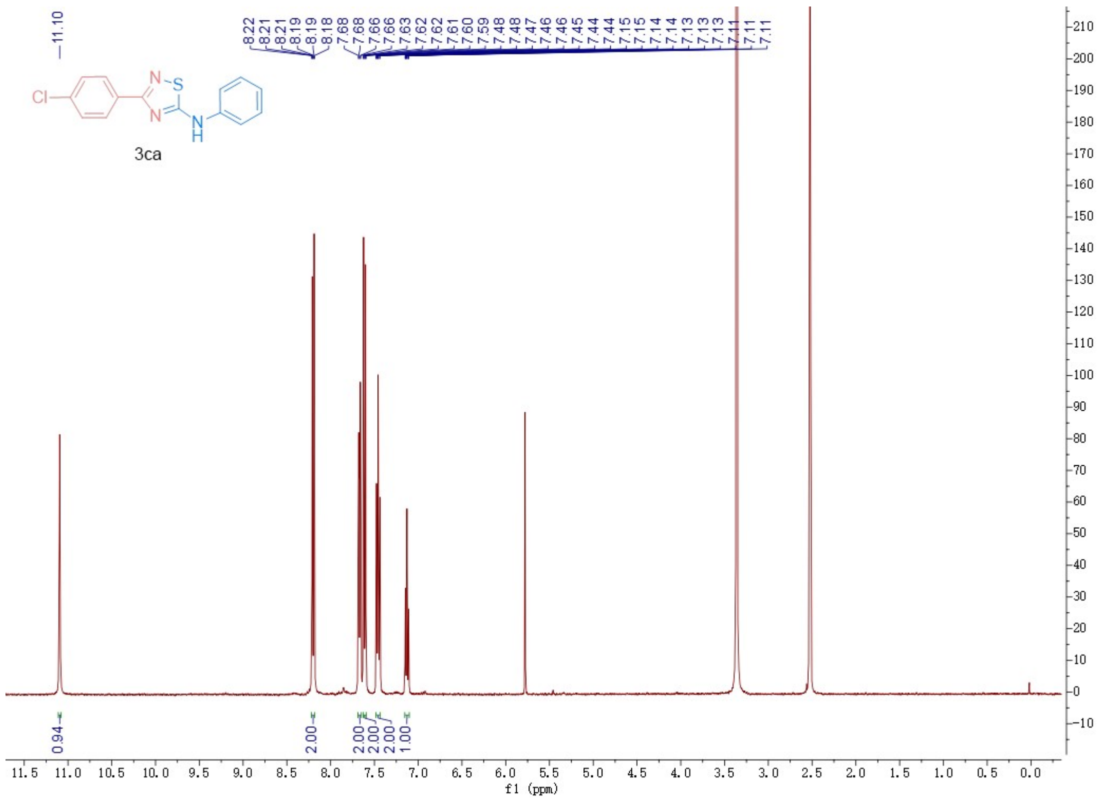
3ca

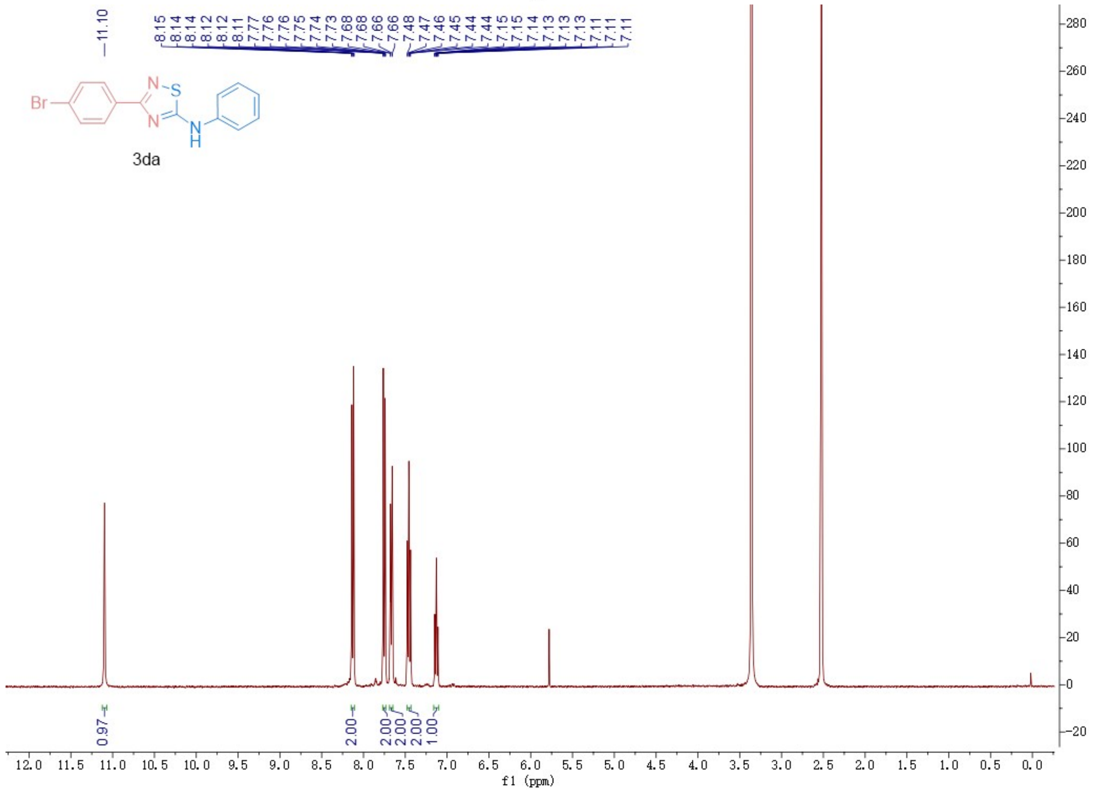
3da

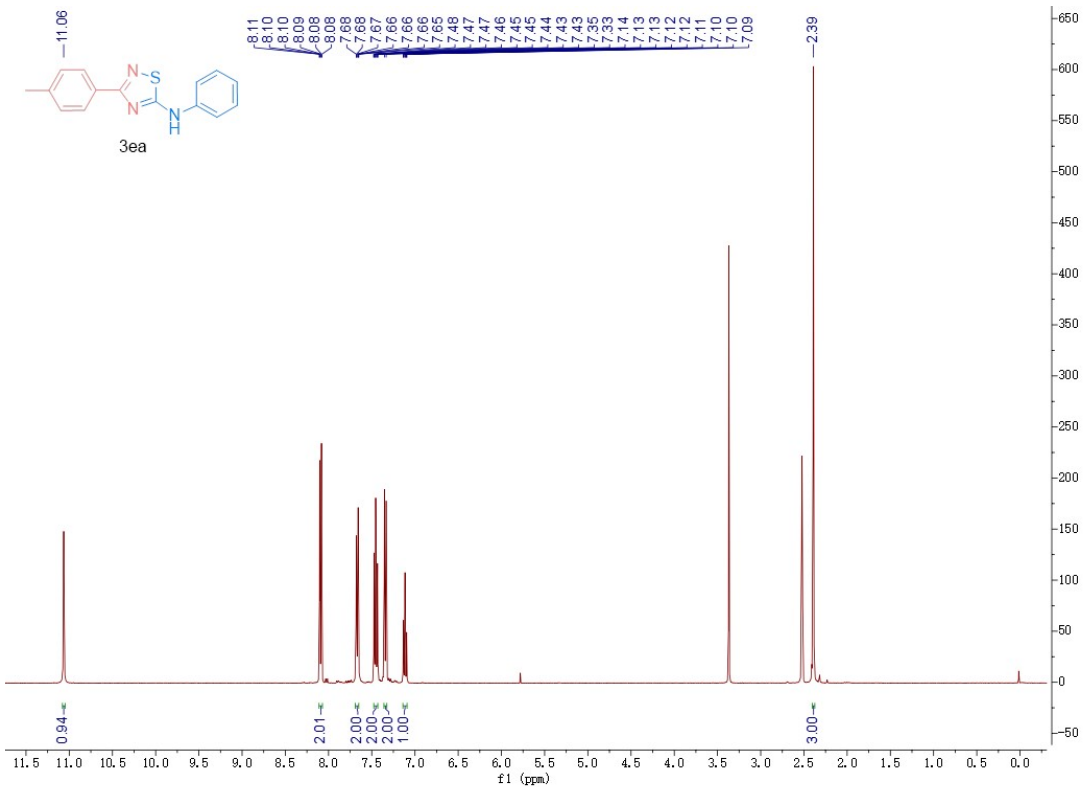

3ea

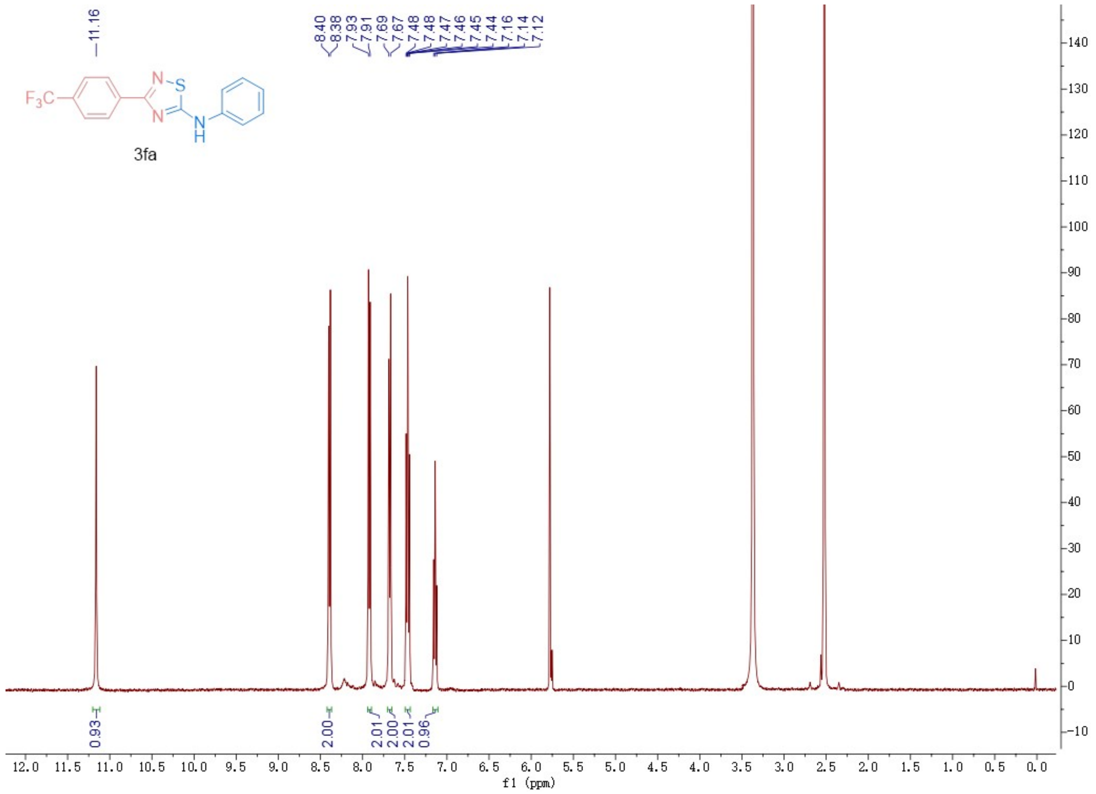

3fa

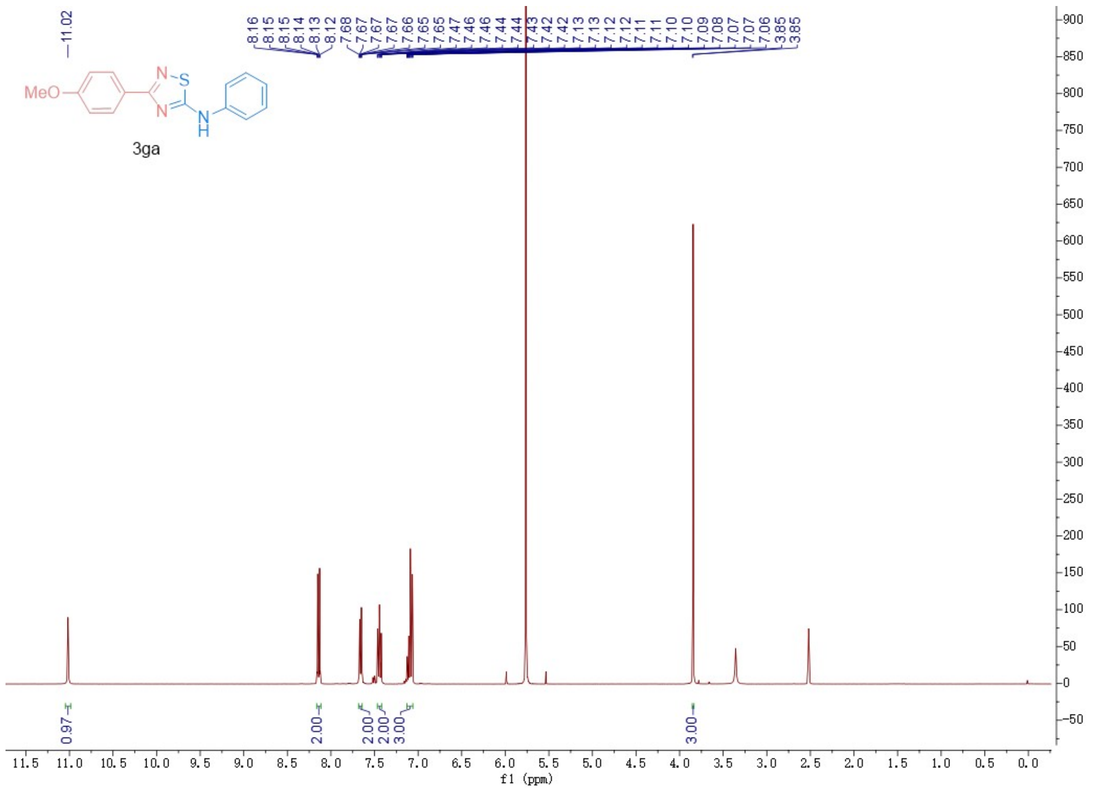

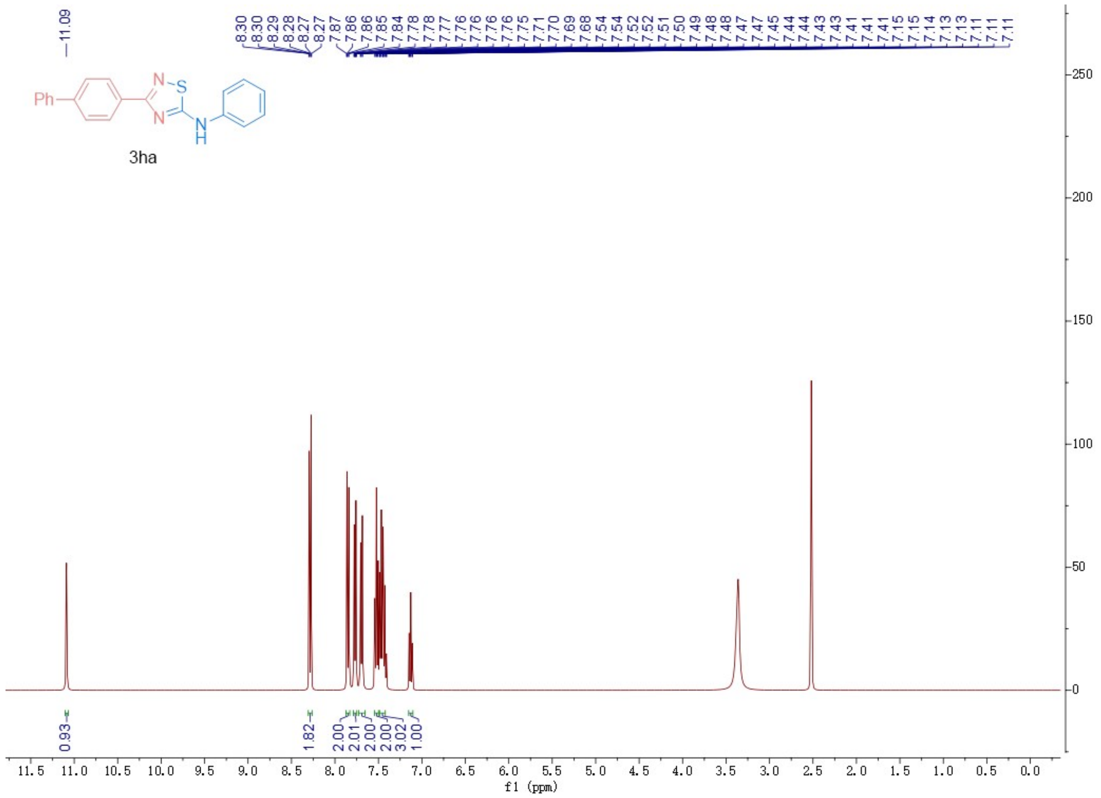

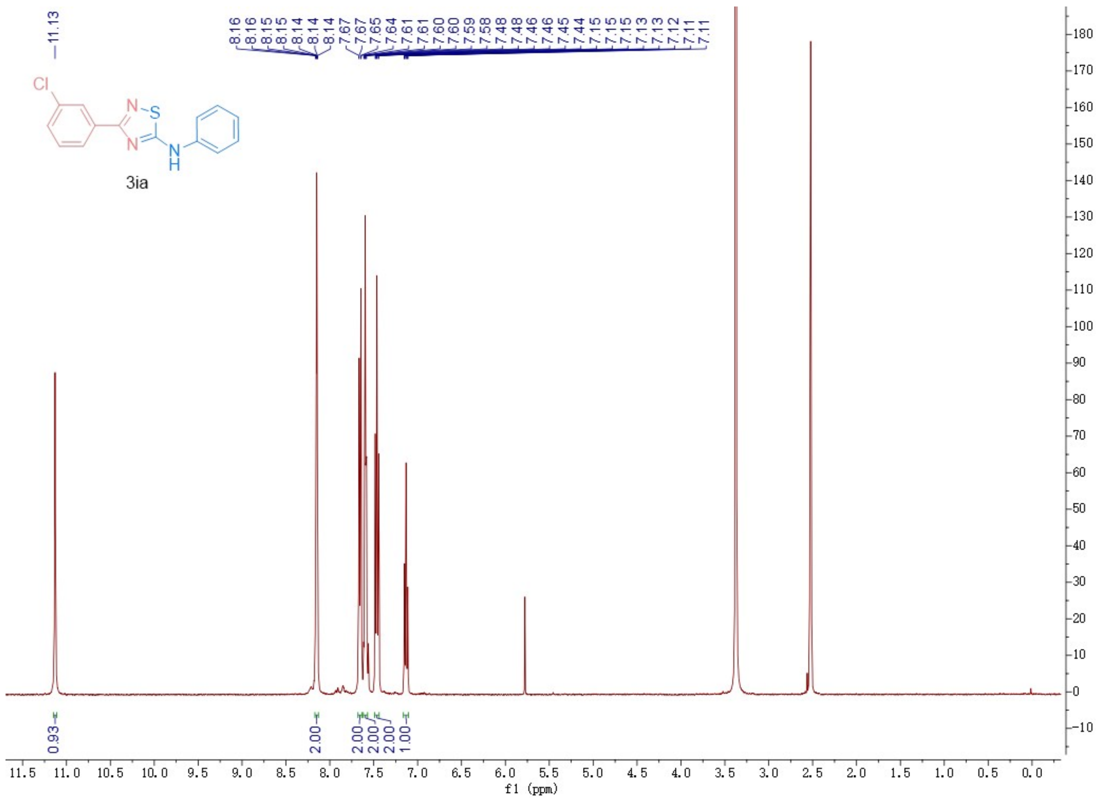

3ia

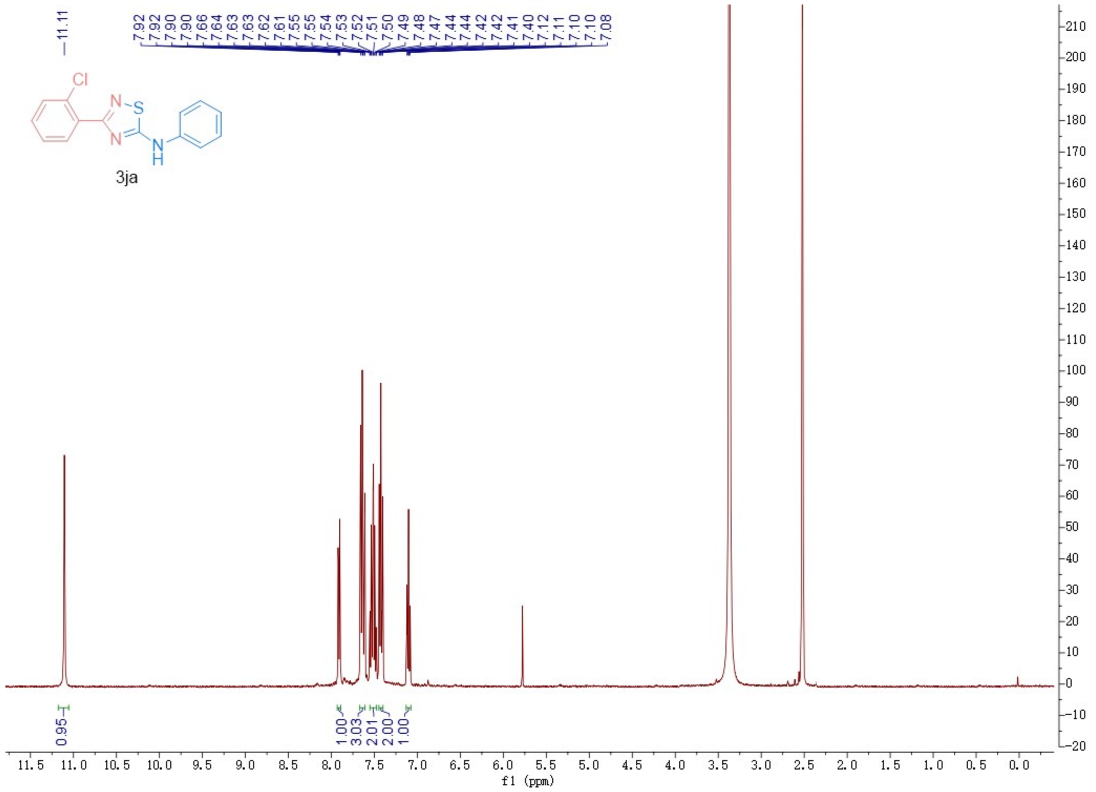

3ja

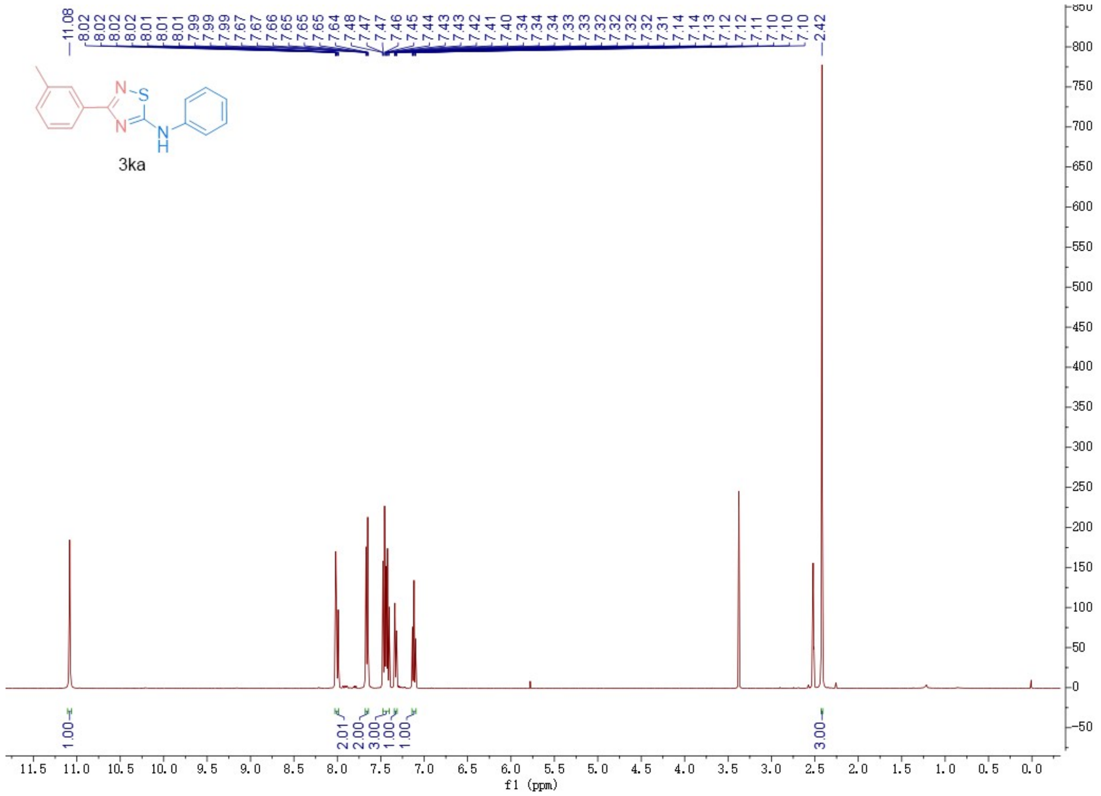

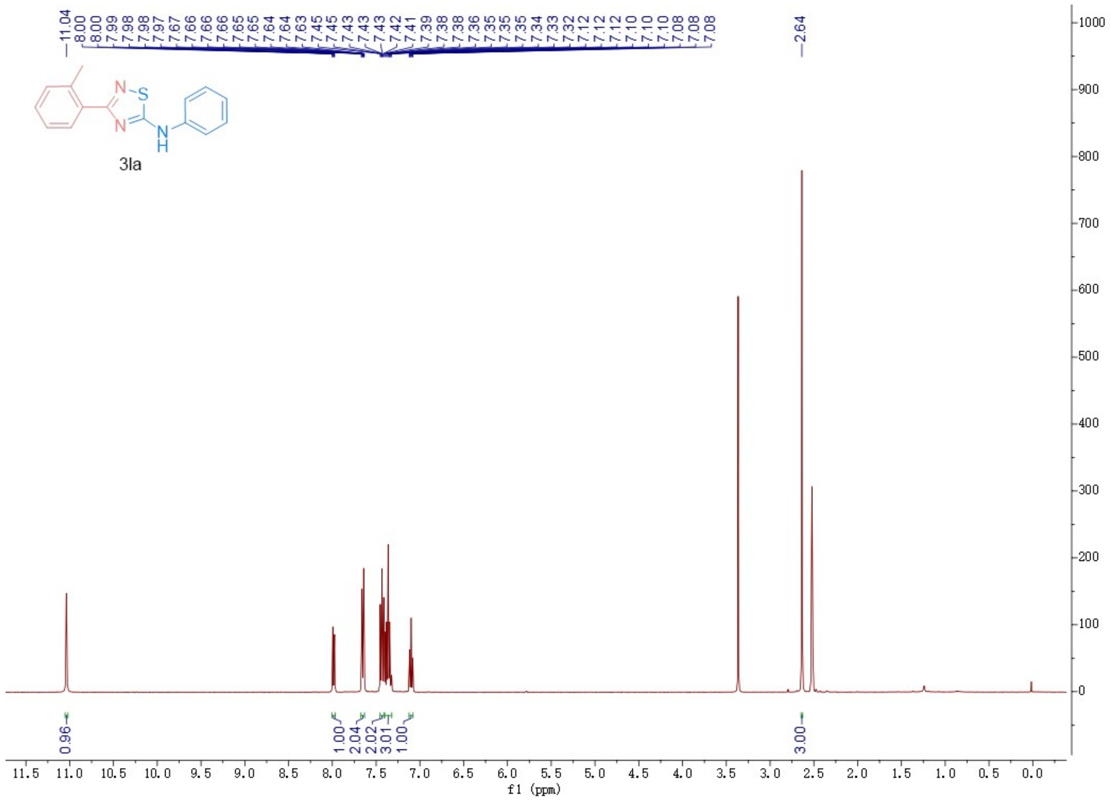

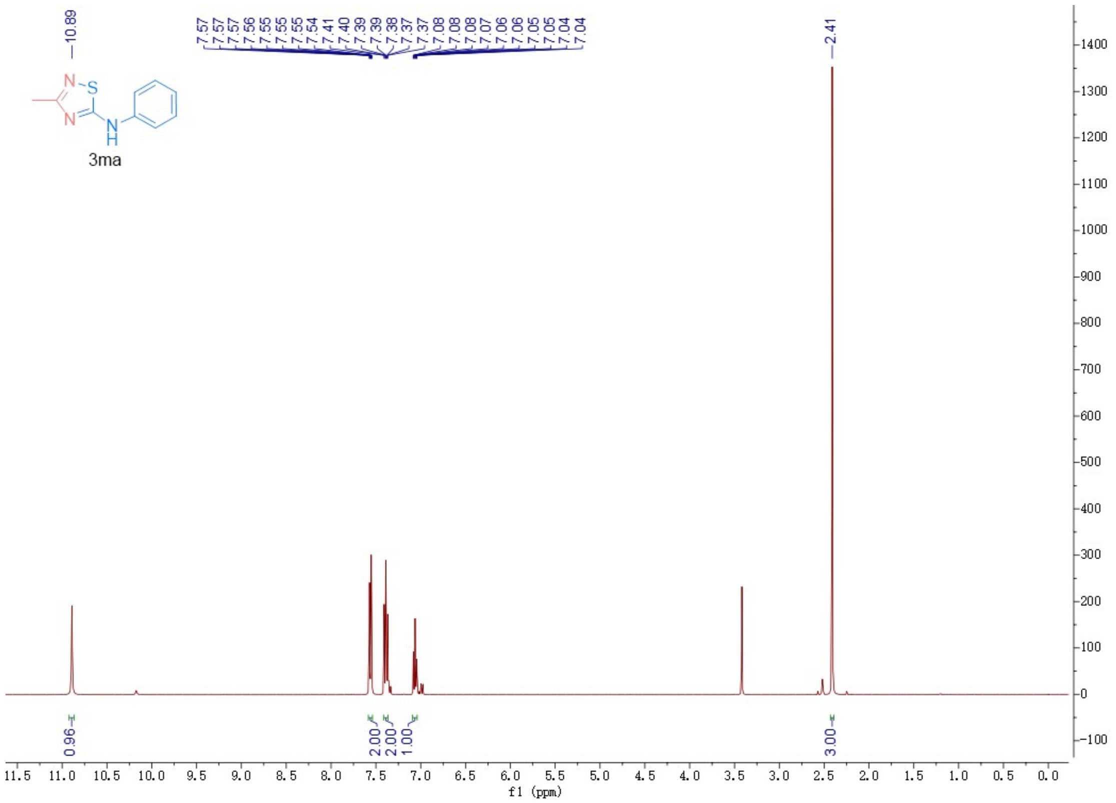
3ma
f1 (ppm)

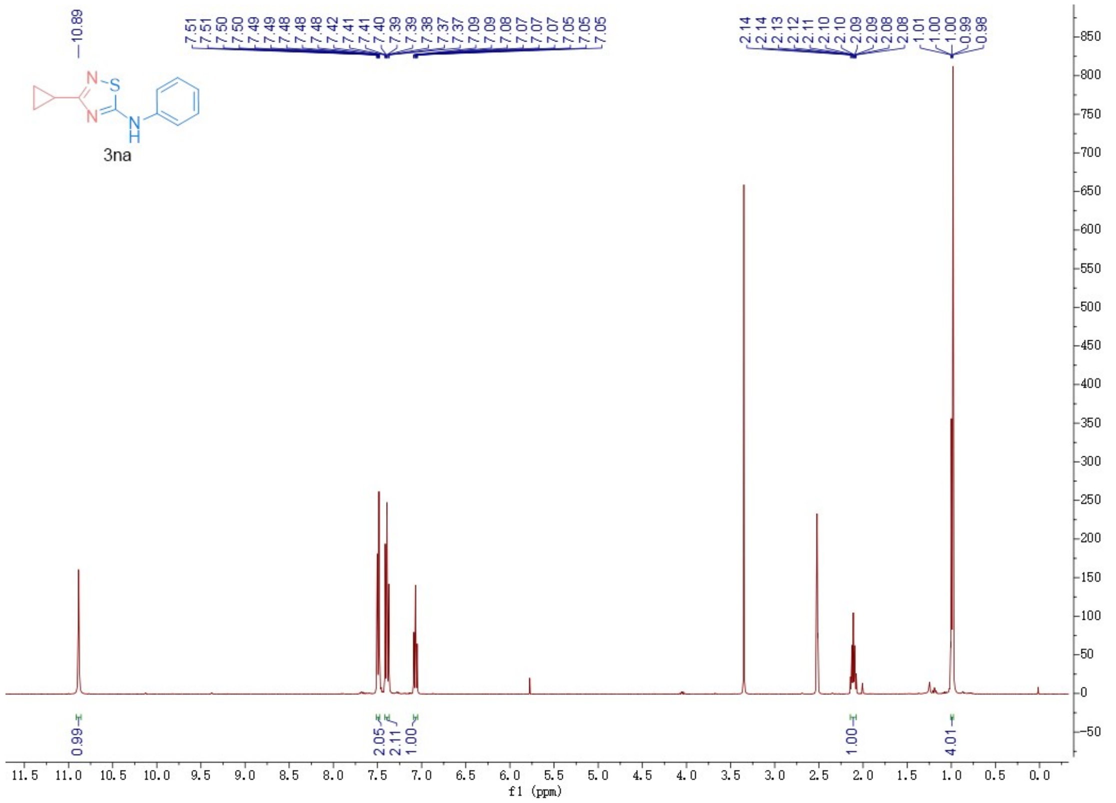
3na
f1 (ppm)

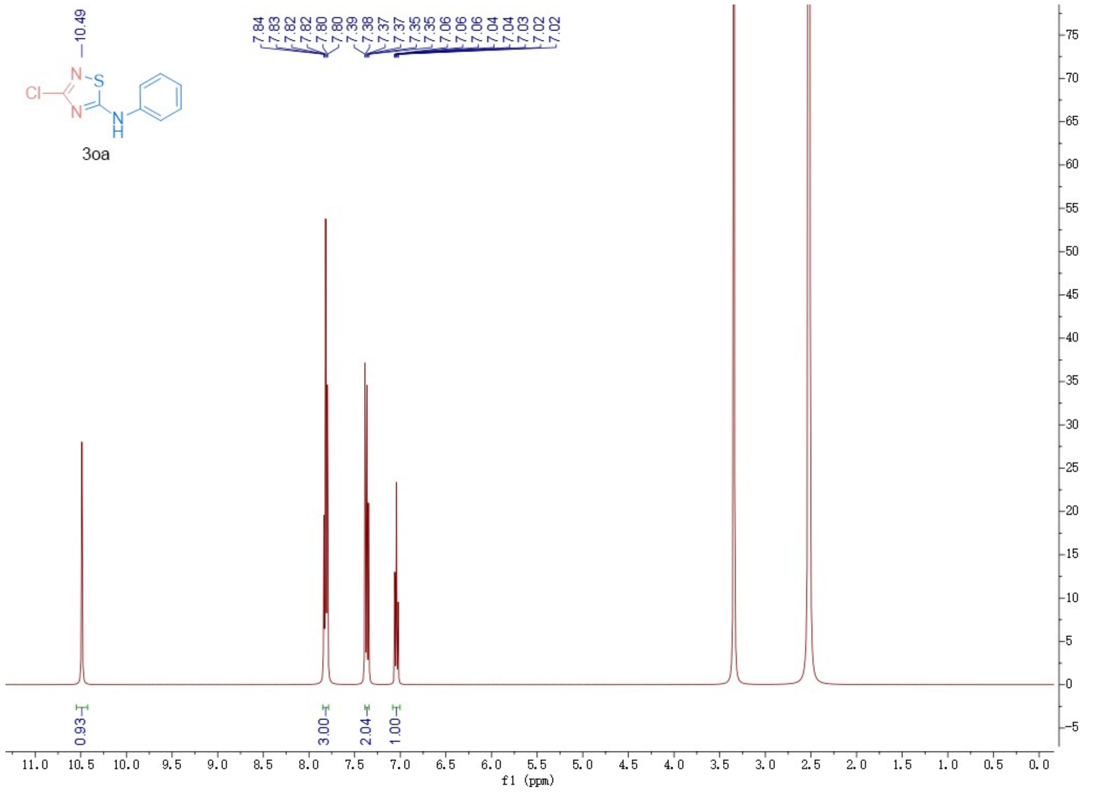

3oa
f1 (ppm)

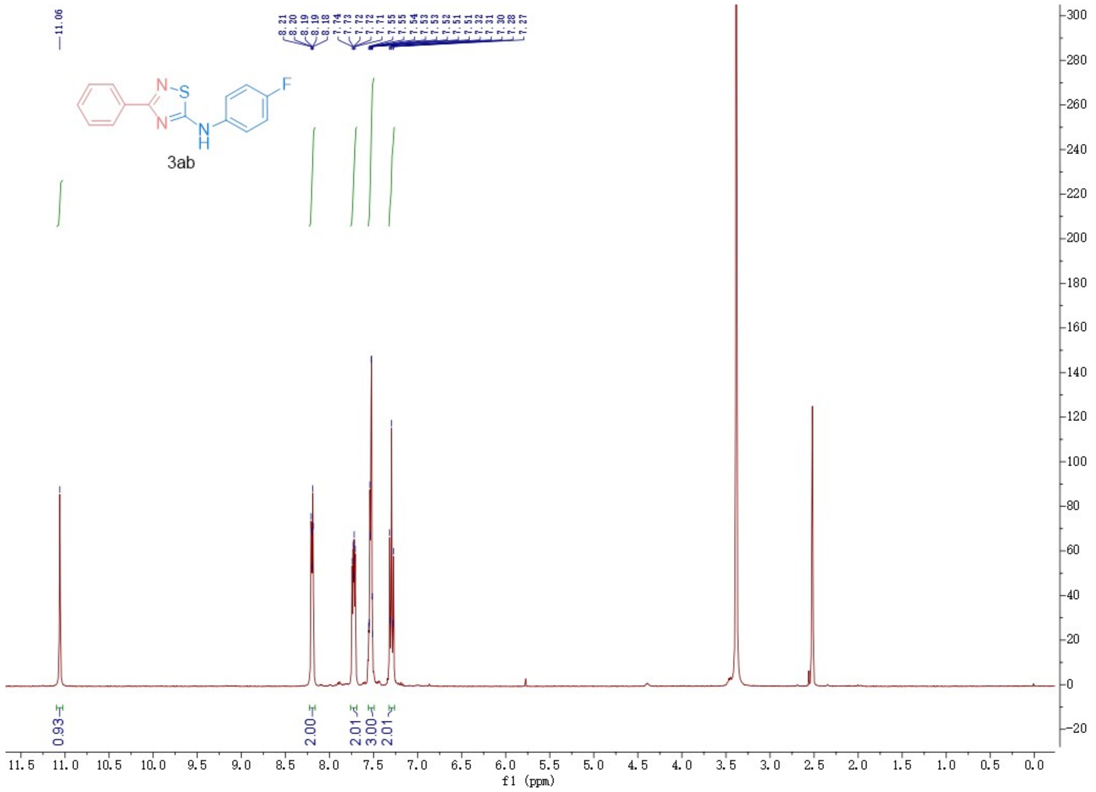

3ab
f1 (ppm)

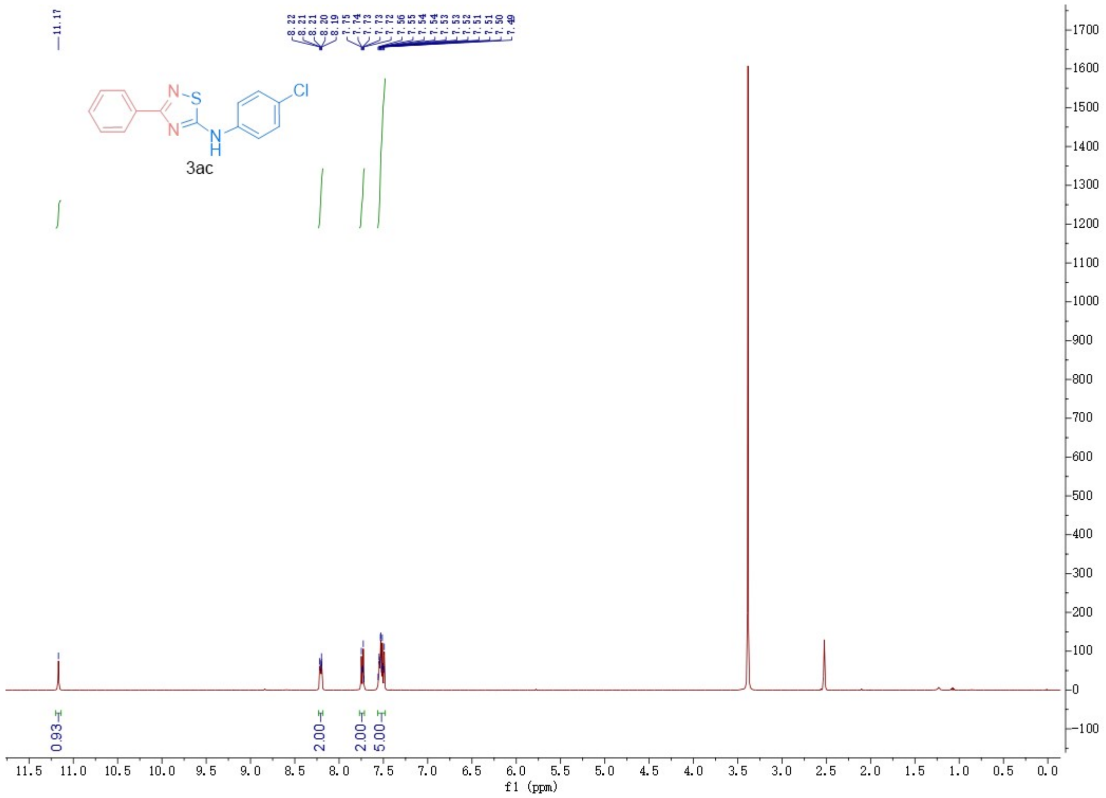
3ac
f1 (ppm)

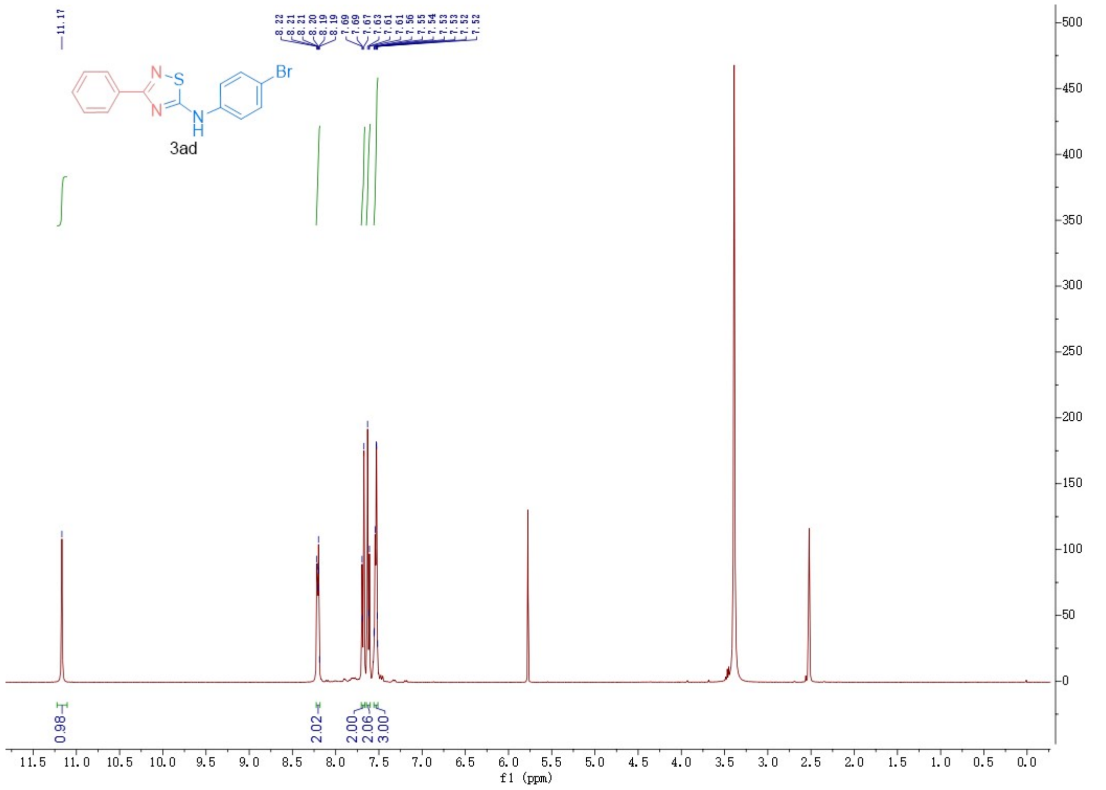
3ad
f1 (ppm)

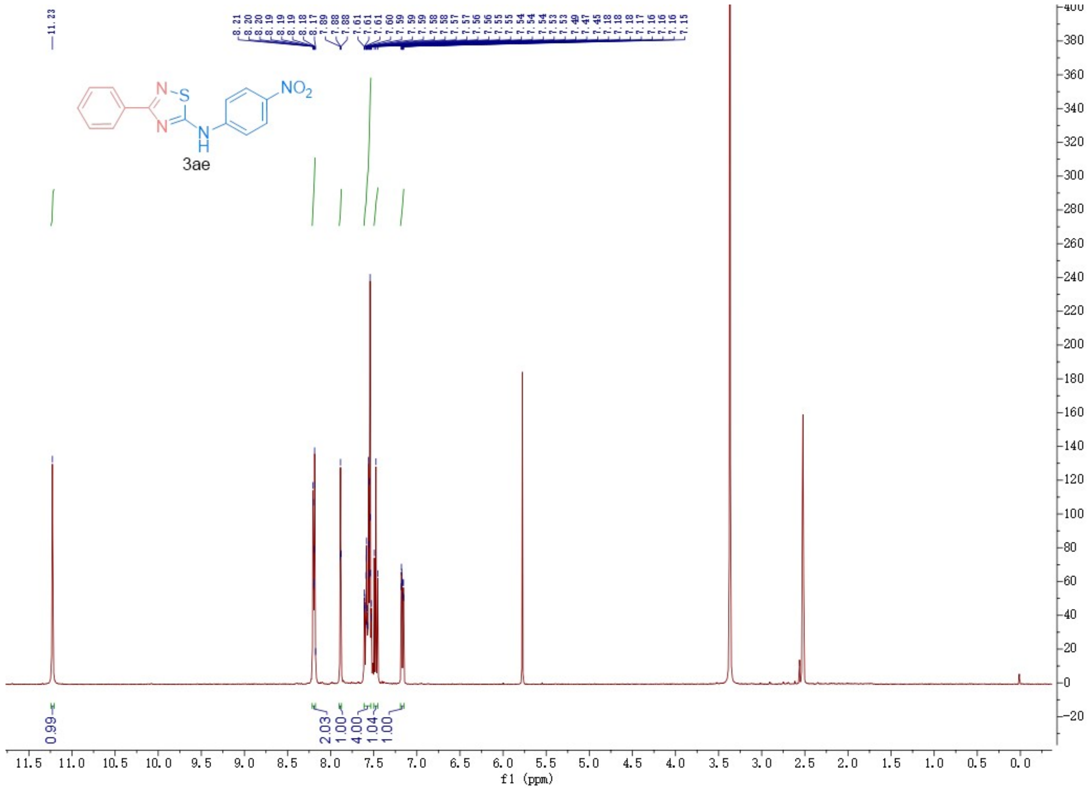

NO2
N
H
3ae
f1 (ppm)

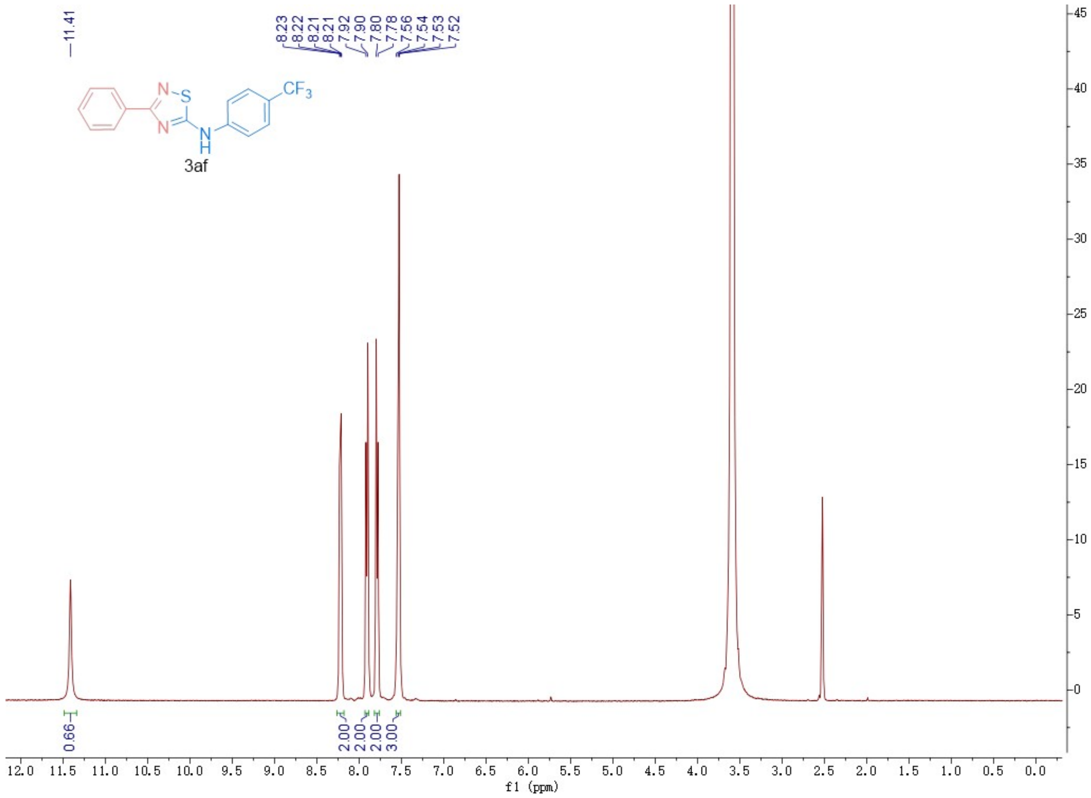

CF3
N
H
3af
f1 (ppm)

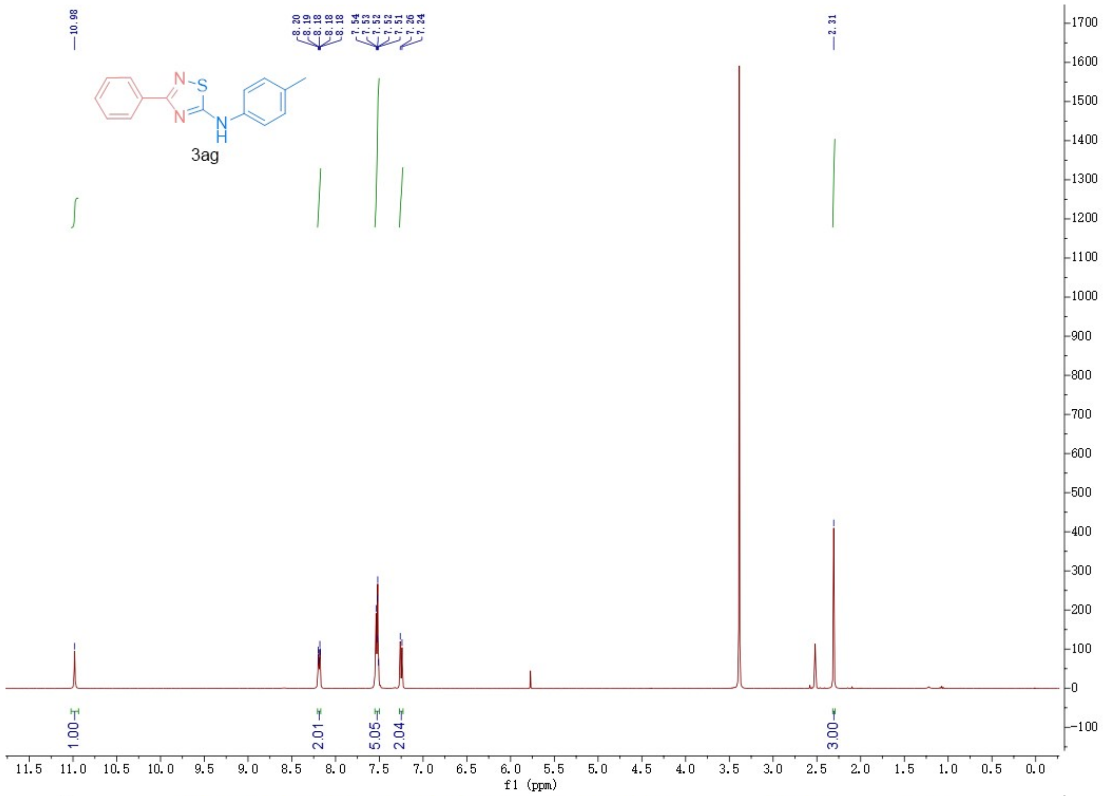
10.98
8.20 8.19 8.18 8.18 8.18
7.54 7.53 7.52 7.52 7.51 7.26 7.24
2.31
N S N H
3ag
1.00
2.01
5.05
2.04
3.00
f1 (ppm)

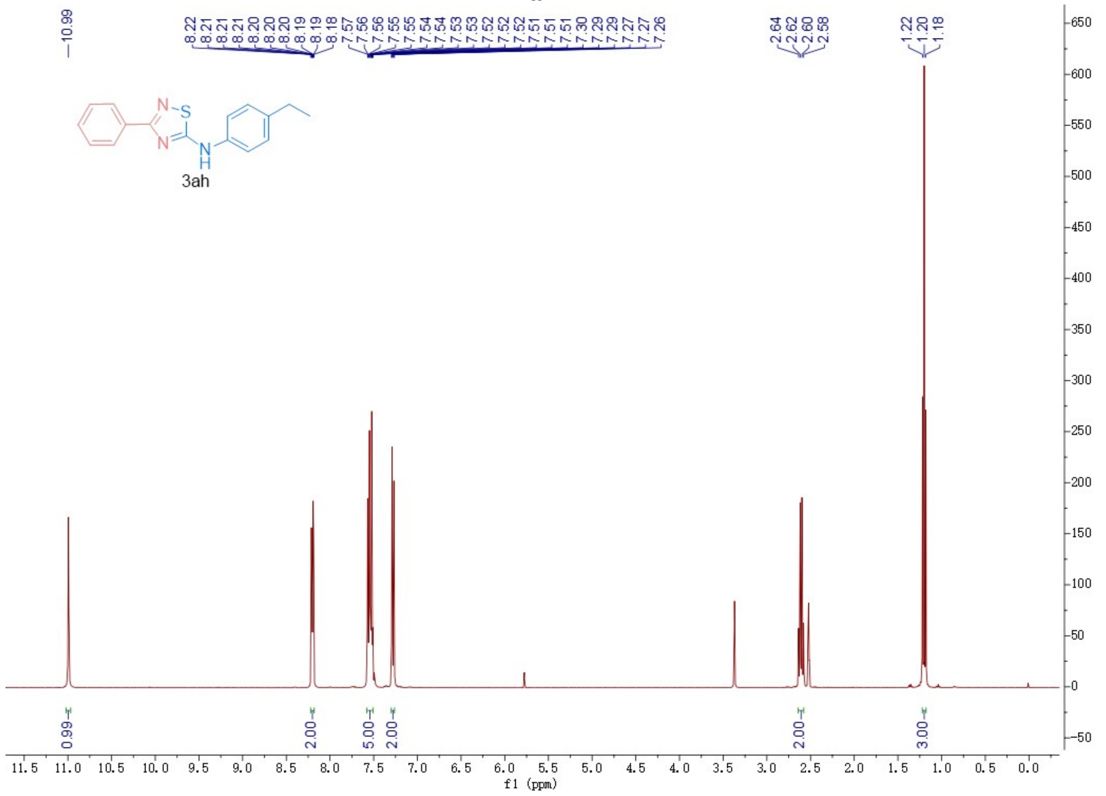
10.99
8.22 8.21 8.21 8.20 8.20 8.19 8.19 8.18 7.57 7.56 7.55 7.55 7.54 7.54 7.53 7.53 7.52 7.52 7.52 7.51 7.51 7.51 7.30 7.29 7.27 7.27 7.26
2.64 2.62 2.60 2.58
1.22 1.20 1.18
N S N H
3ah
0.99
2.00
5.00
2.00
2.00
3.00
f1 (ppm)

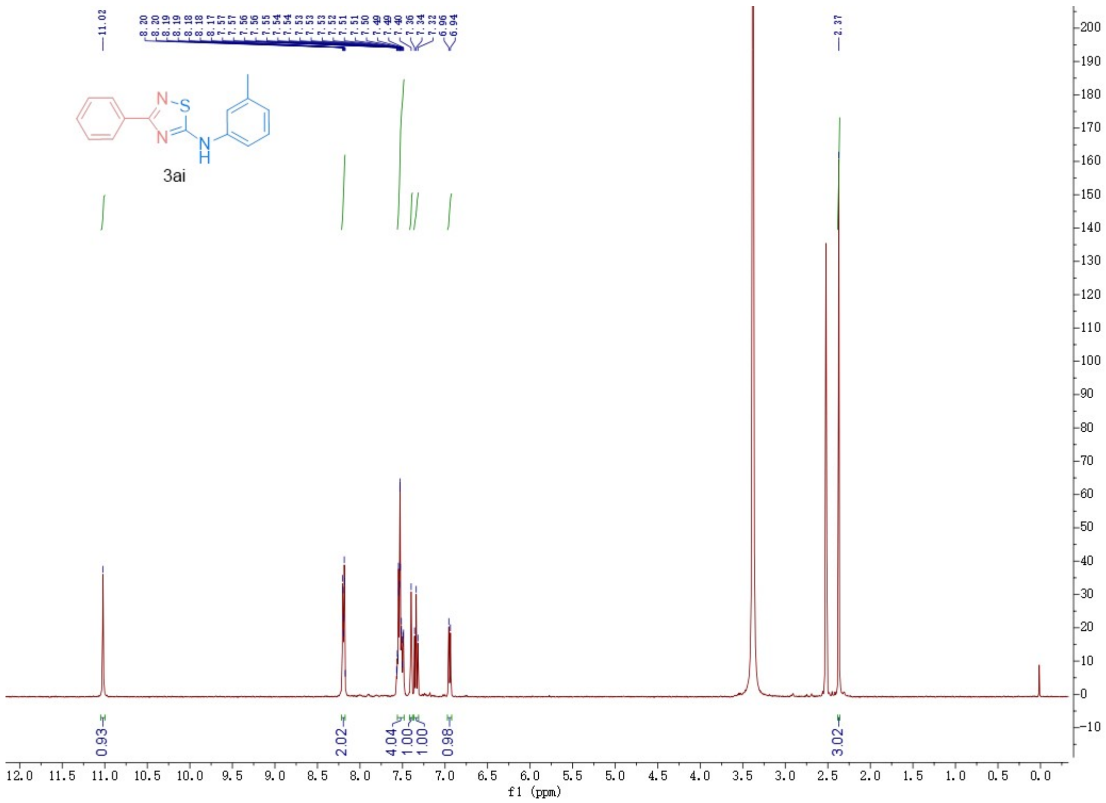
3ai
f1 (ppm)

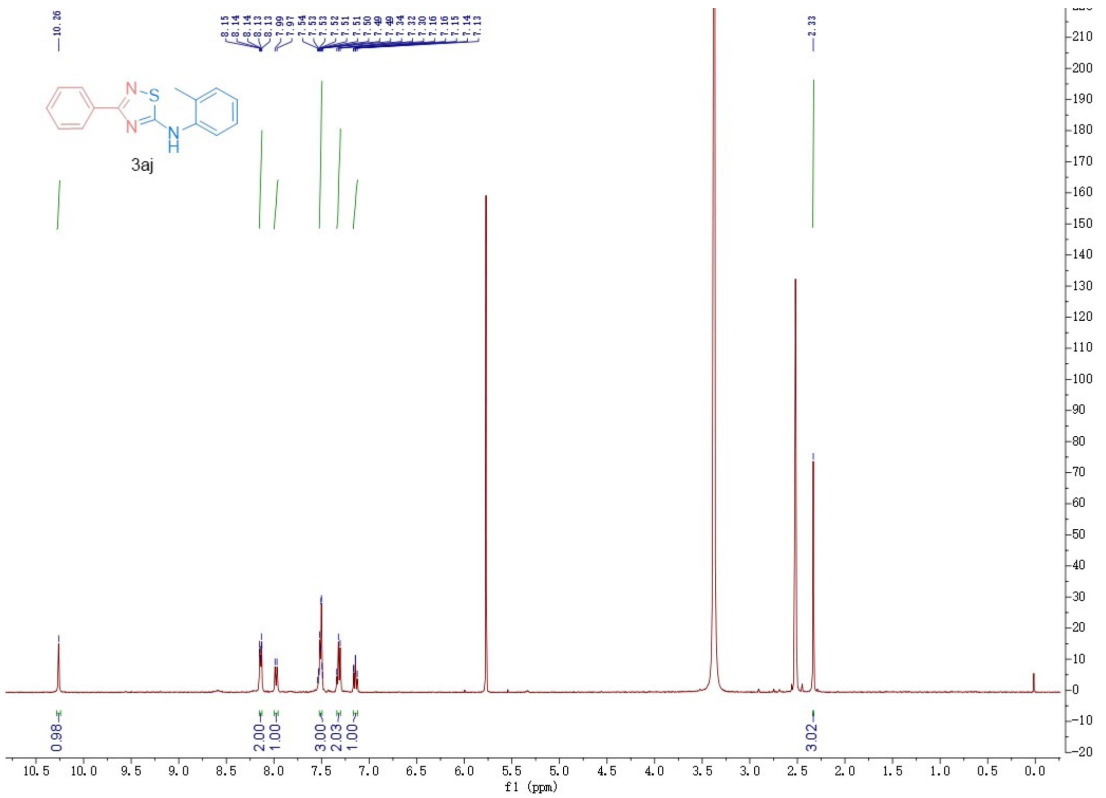
3aj
f1 (ppm)

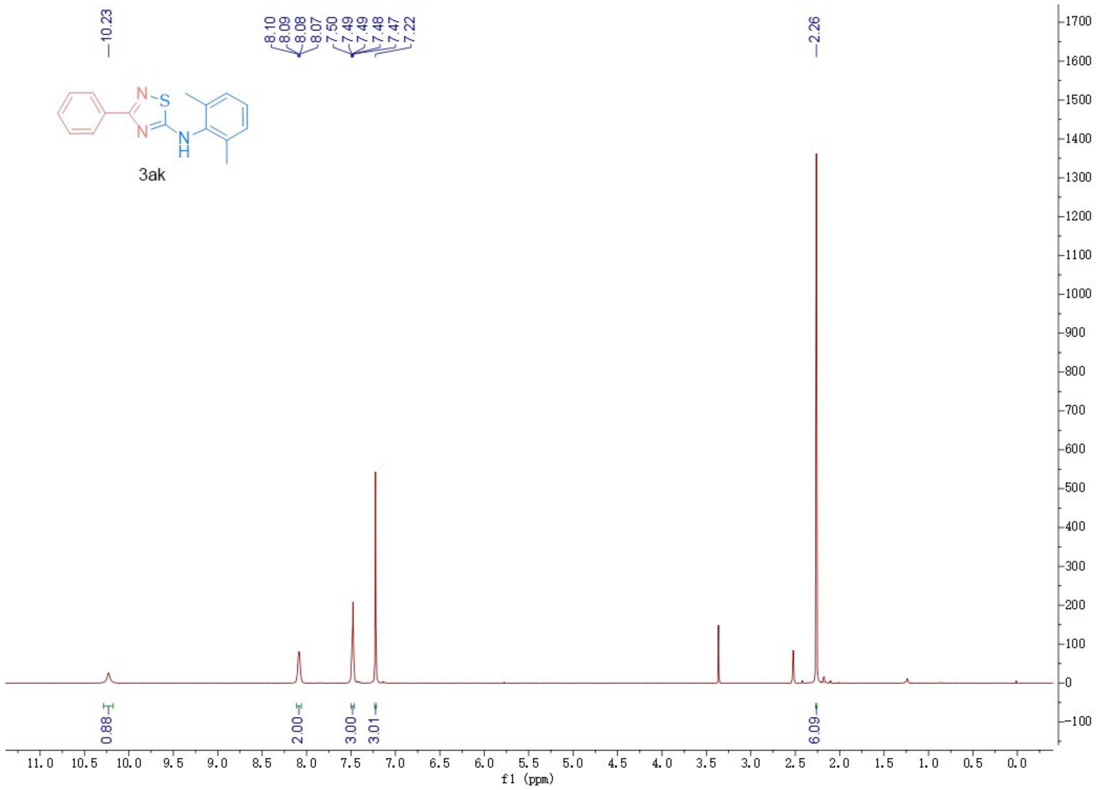
10.23
8.10
8.09
8.08
8.07
7.50
7.49
7.49
7.48
7.47
7.22
2.26
3ak
0.88
2.00
3.00
3.01
6.09
f1 (ppm)

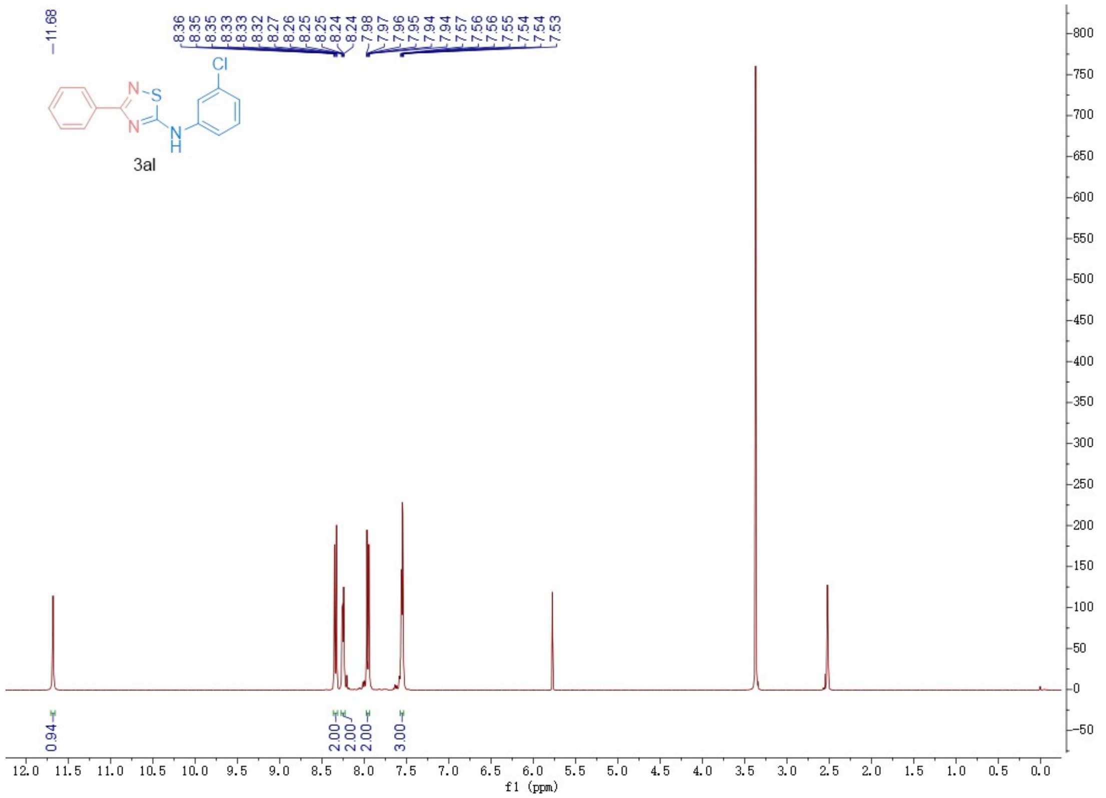
11.68
8.36
8.35
8.33
8.33
8.32
8.27
8.26
8.25
8.24
8.24
7.98
7.97
7.96
7.95
7.94
7.94
7.57
7.56
7.56
7.55
7.54
7.54
7.53
Cl
3al
0.94
2.00
2.00
2.00
3.00
f1 (ppm)

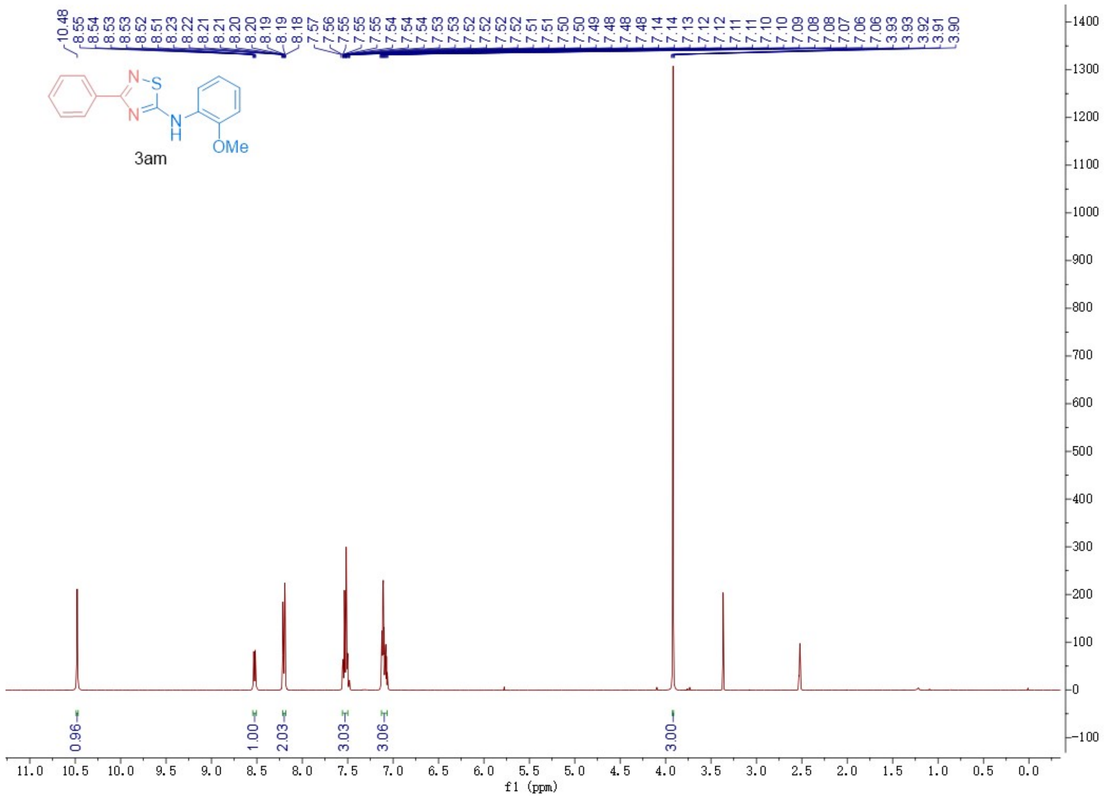
3am
OMe
f1 (ppm)

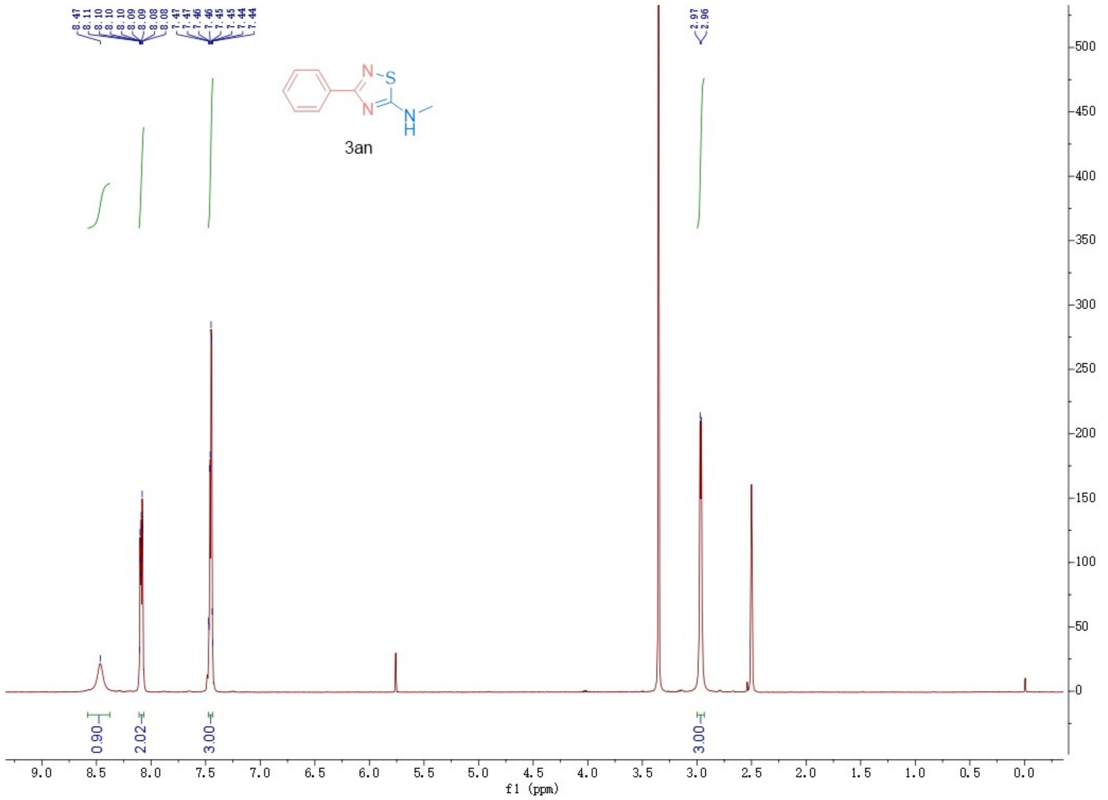
3an
f1 (ppm)

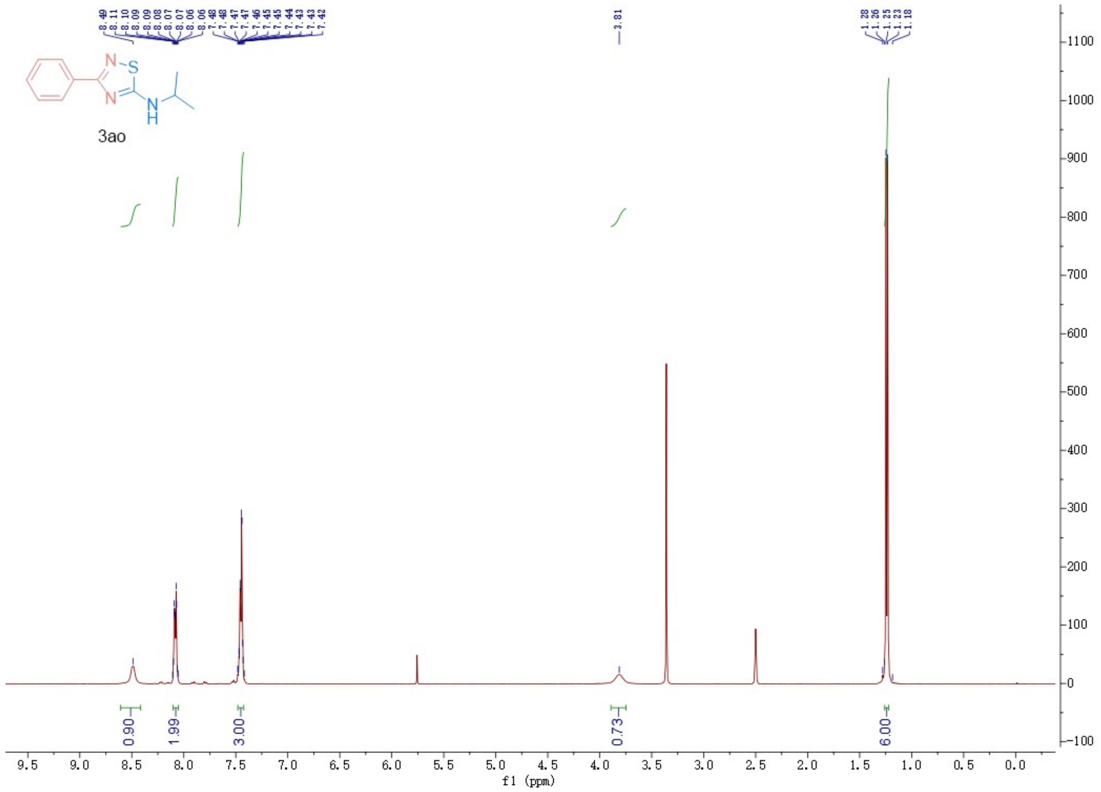

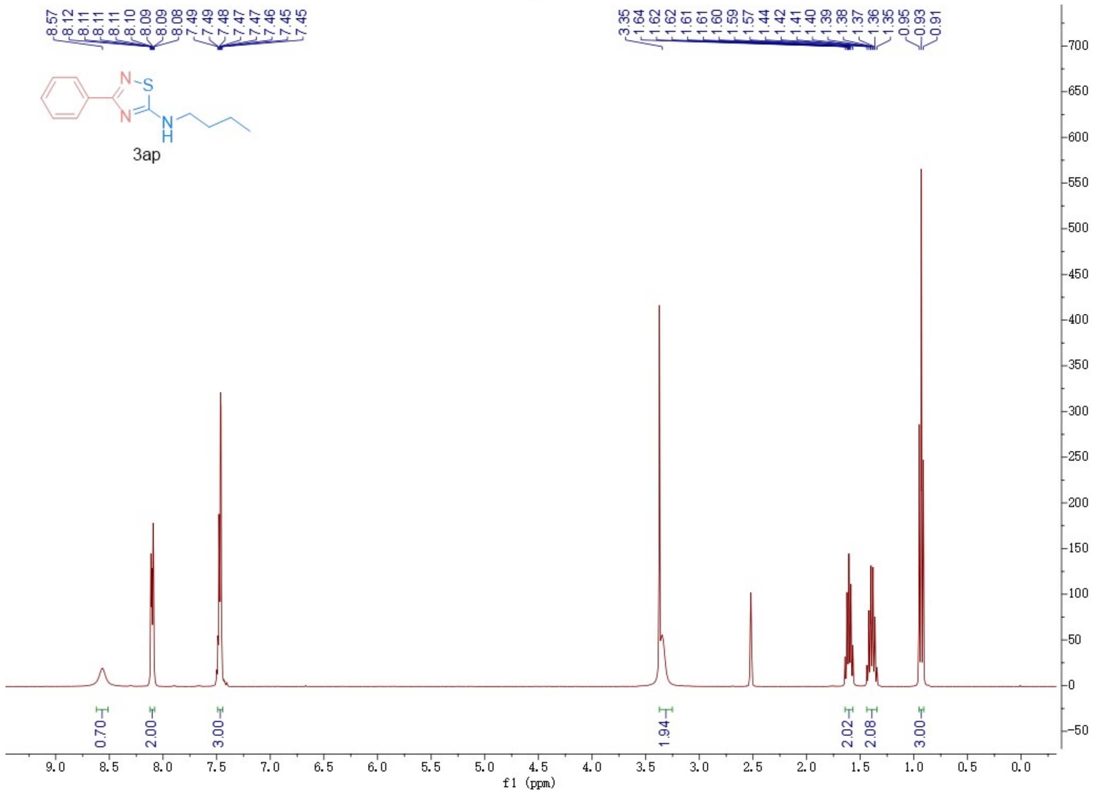

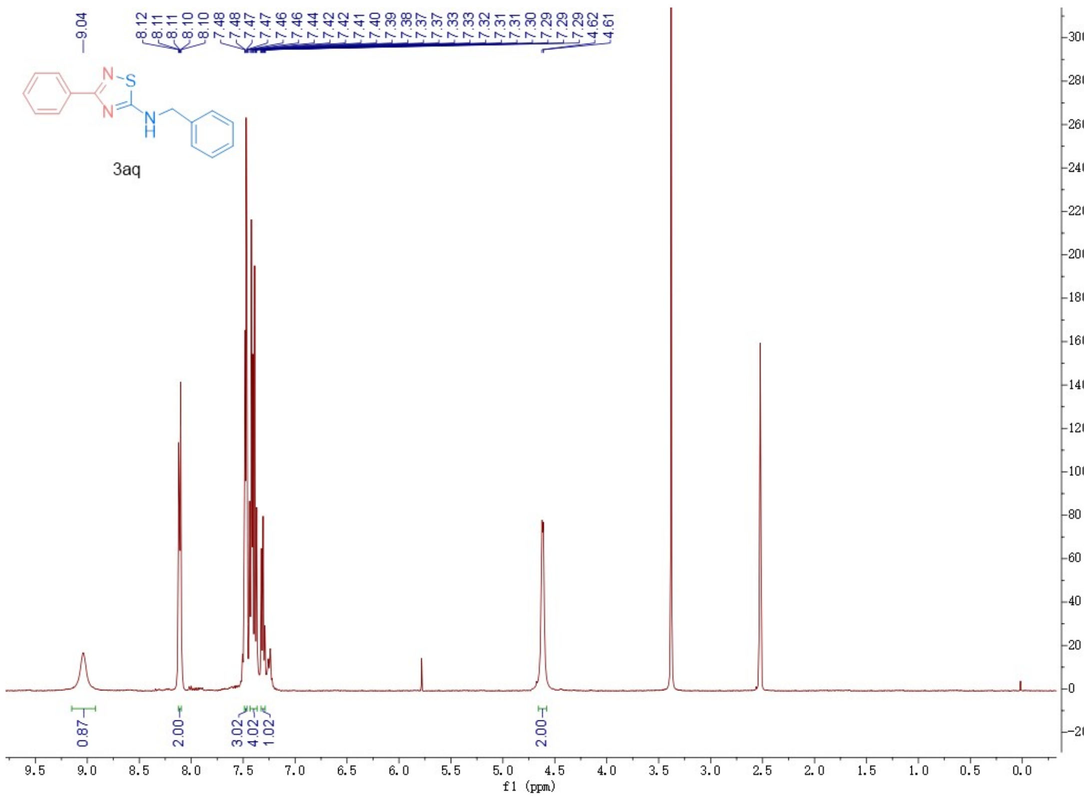

9.04
8.12
8.11
8.11
8.10
7.48
7.48
7.47
7.47
7.46
7.46
7.44
7.42
7.42
7.41
7.40
7.39
7.38
7.37
7.37
7.33
7.33
7.32
7.31
7.31
7.30
7.29
7.29
7.29
4.62
4.61
N
S
N
N
H
3aq
0.87
2.00
3.02
4.02
1.02
2.00
f1 (ppm)

^{13}C NMR Spectra of New Products

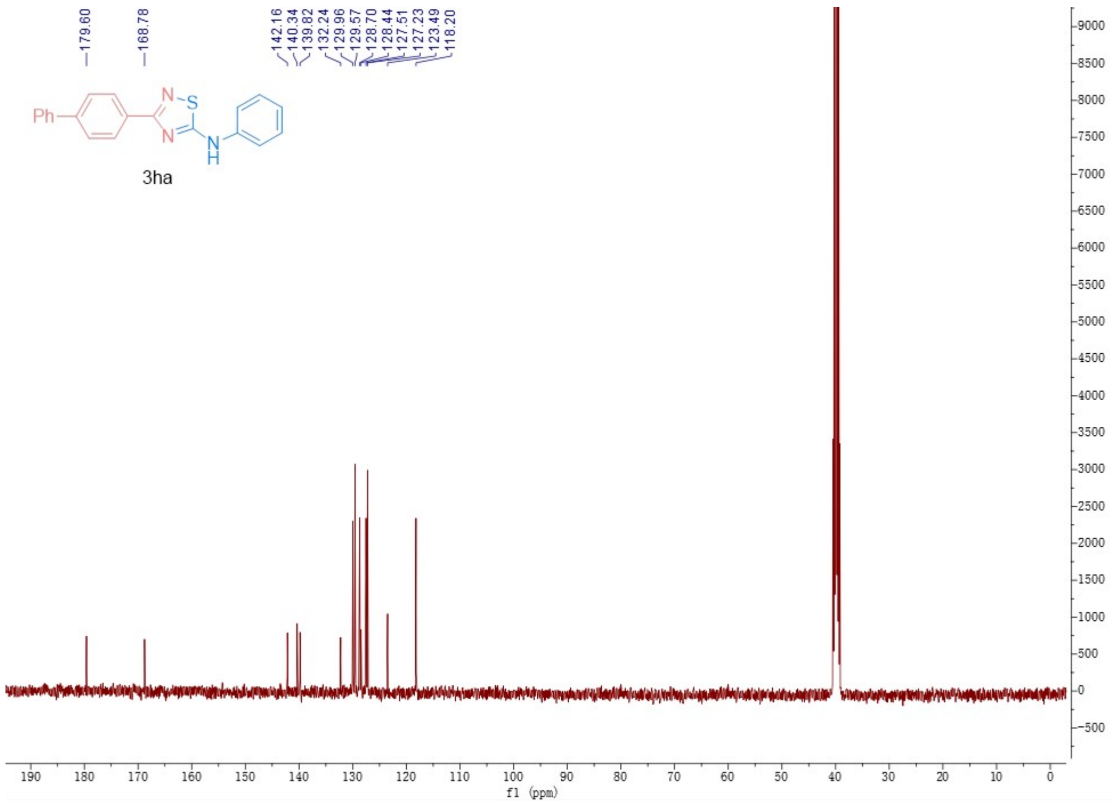

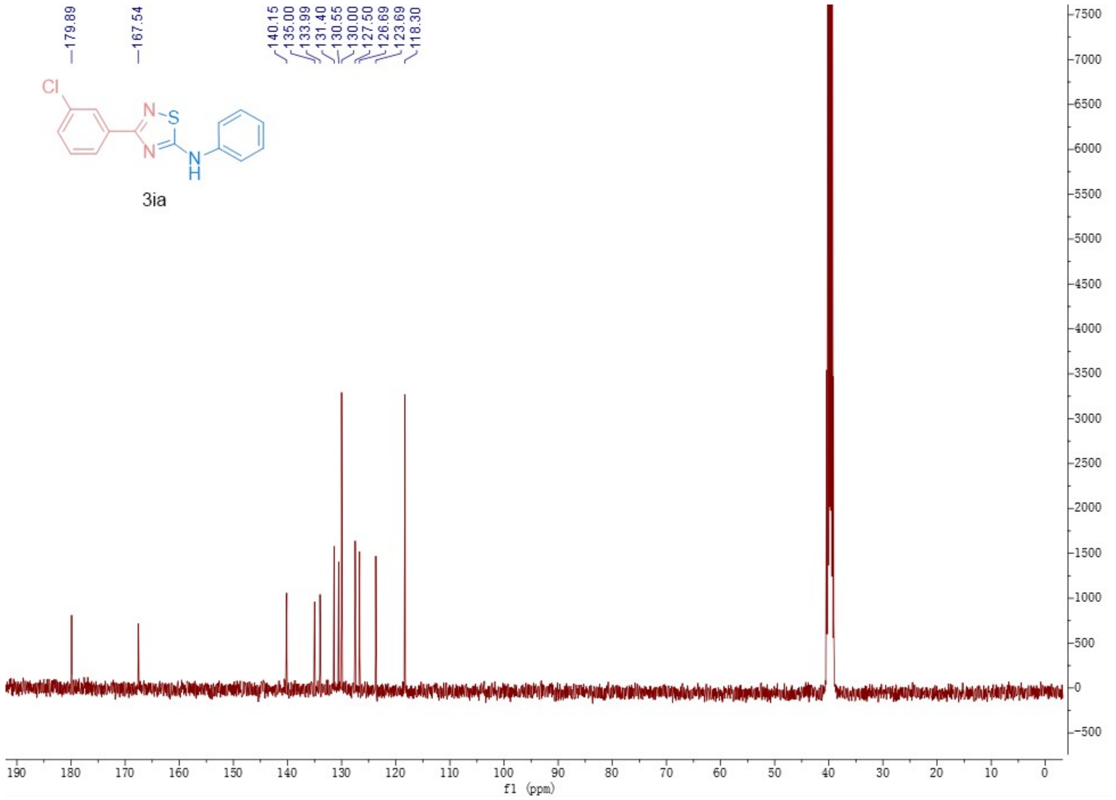

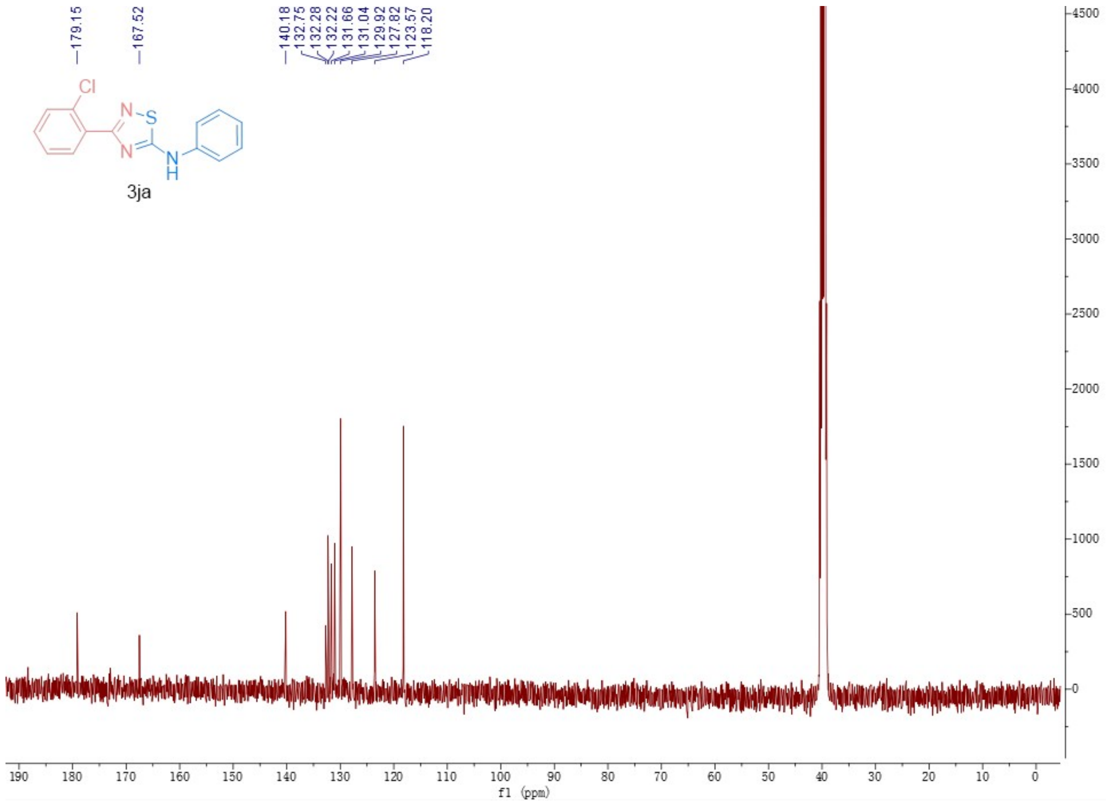

179.15
167.52
140.18
132.75
132.28
132.22
131.66
131.04
129.92
127.82
123.57
118.20
Cl
N
S
N
N
H
3ja
f1 (ppm)

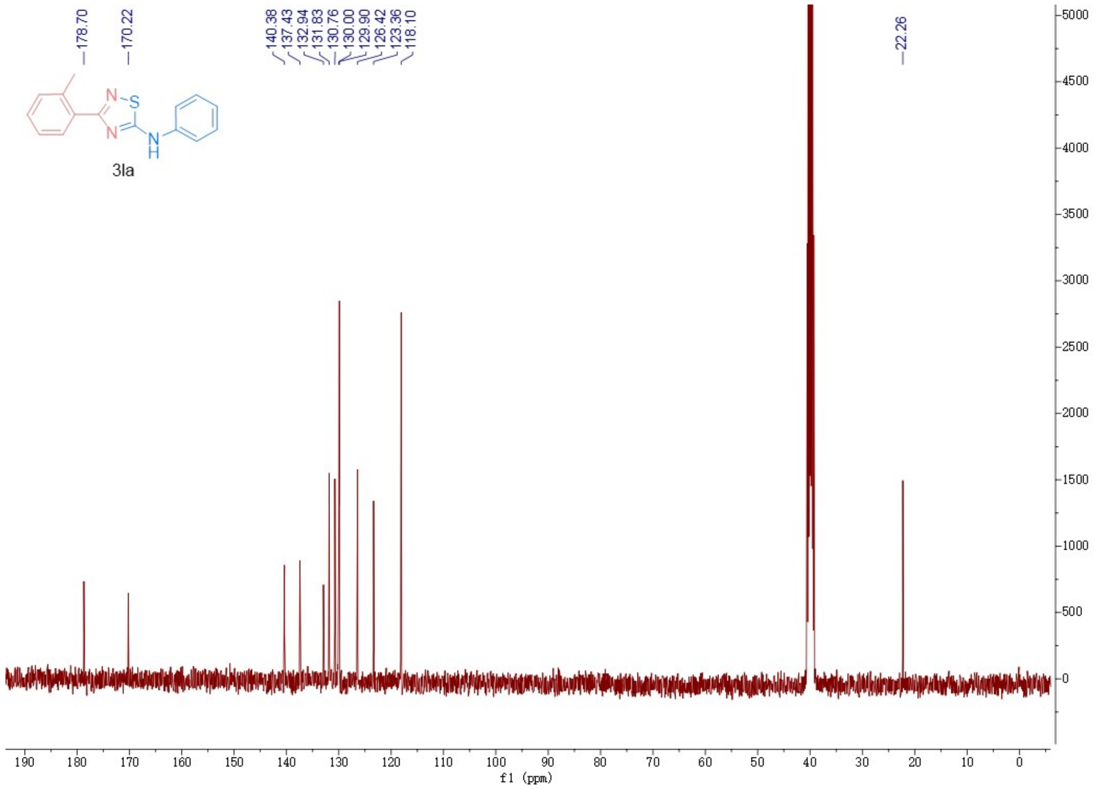

178.70
170.22
140.38
137.43
132.94
131.83
130.76
130.00
129.90
126.42
123.36
118.10
22.26
N
S
N
N
H
3la
f1 (ppm)

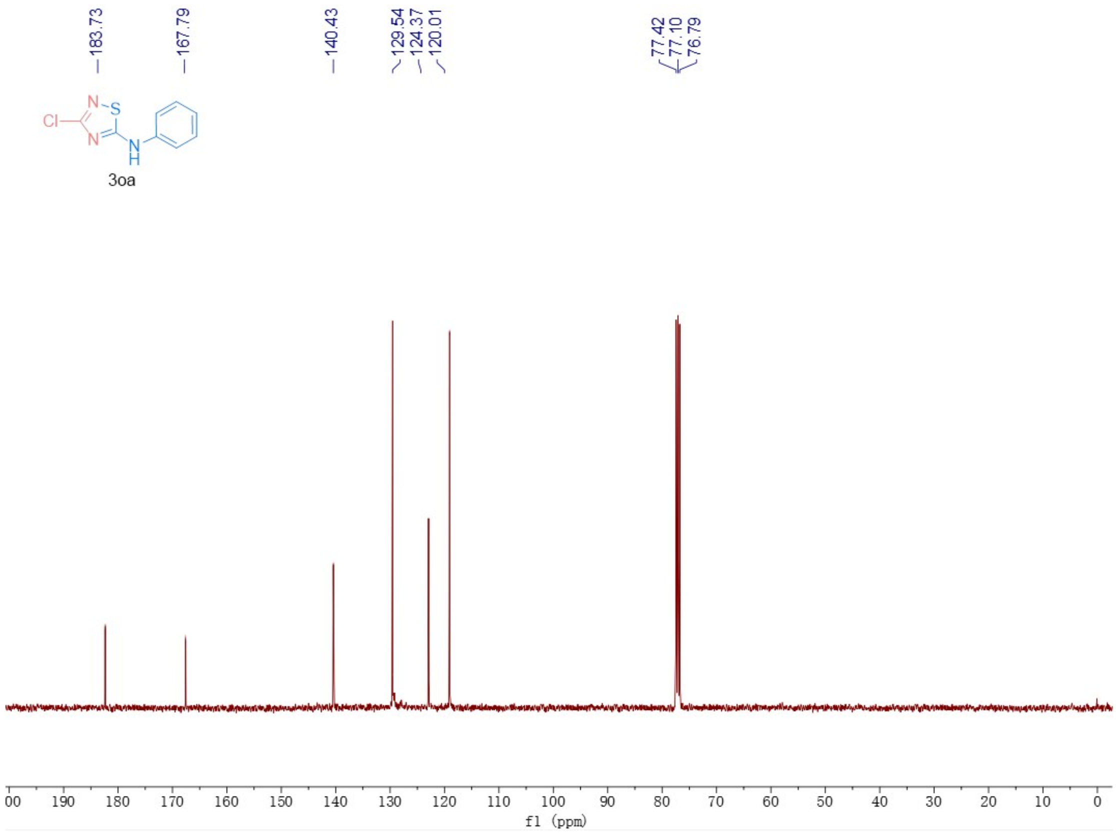
183.73
167.79
140.43
129.54
124.37
120.01
77.42
77.10
76.79
Cl
N
S
N
N
H
3oa
00
190
180
170
160
150
140
130
120
110
100
90
80
70
60
50
40
30
20
10
0
f1 (ppm)

Reference

1 F. P. Guengerich, M. V. Martin, C. D. Sohl, Q. Cheng, *Nat. Protoc.* 2009, **4**, 1245–1251.
2 K. L. Dikshit, D. A. Webster, *Gene*, 1988, **70**, 377-386.
3 A. Waterhouse, M. Bertoni, S. Bienert, G. Studer, G. Tauriello, R. Gumienny, F. T. Heer, T. A P. de Beer, C. Rempfer, L. Bordoli, R. Lepore and T. Schwede, *Nucleic Acids Res.*, 2018, **46**, W296-W303.
4 E. F. Pettersen, T. D. Goddard, C. C. Huang, G. S. Couch, D. M. Greenblatt, E. C. Meng and T. E. Ferrin, *J. Comput. Chem.*, 2004, **25**, 1605-1612.
5 O. Trott and A. J. Olson, *J. Comput. Chem.*, 2010, **31**, 455-461.
6 A. S. Inc, *Accelrys*, 2016.
7 L. Yang, L. Song, S. Tang, L. Li, H. Li, B. Yuan and G. Yang, *Eur. J. Org. Chem.*, 2019, **2019**, 1281-1285.
8 B. Wang, Y. Meng, Y. Zhou, L. Ren, J. Wu, W. Yu and J. Chang, *J. Org. Chem.*, 2017, **82**, 5898-5903.
9 N. Jatangi, N. Tumula, R. K. Palakodety and M. Nakka, *J. Org. Chem.*, 2018, **83**, 5715-5723.
10 Z. Yang, J. Zhang, L. Hu, L. Li, K. Liu, T. Yang and C. Zhou, *J. Org. Chem.*, 2020, **85**, 3358-3363.
11 J. Yuan, Q. Xia, W. Zhu, C. Wu, B. Wang, B. Liu, X. Yang, Y. Xu and H. Xu, *ChemPhotoChem*, 2020, **4**, 445-450.
12 W. Yu, Y. Huang, J. Li, X. Tang, W. Wu and H. Jiang, *J. Org. Chem.*, 2018, **83**, 9334-9343.
13 A. Mariappan, K. Rajaguru, N. Merukan Chola, S. Muthusubramanian and N. Bhuvanesh, *J. Org. Chem.*, 2016, **81**, 6573-6579.
14 X.-T. Cao, Z.-L. Zheng, J. Liu, Y.-H. Hu, H.-Y. Yu, S. Cai and G. Wang, *Adv. Synth. Catal.*, 2022, **364**, 689-694.
15 Z. Xiong, Q. Zhong, S. Sheng and J. Chen, *ARKIVOC*, 2021, **2021**, 340-348.
16 J.-X. Lin, G.-H. Liu, L.-Q. Liu, Y.-C. Wang and Y. He, *J. Org. Chem.*, 2024, **89**, 101-110.
17 J. Y. Park, I. A. Ryu, J. H. Park, D. C. Ha and Y.-D. Gong, *Synthesis*, 2009, **2009**, 913-920.
